# Prenatal SMN-dependent defects in translation uncover reversible primary cilia phenotypes in spinal muscular atrophy

**DOI:** 10.1172/jci.insight.192835

**Published:** 2025-09-09

**Authors:** Federica Genovese, Yu-Ting Huang, Anna A.L. Motyl, Martina Paganin, Gaurav Sharma, Ilaria Signoria, Deborah Donzel, Nicole C.H. Lai, Marie Pronot, Rachel A. Kline, Helena Chaytow, Kimberley J. Morris, Kiterie M.E. Faller, Thomas M. Wishart, Ewout J.N. Groen, Michael A. Cousin, Gabriella Viero, Thomas H. Gillingwater

**Affiliations:** 1 Edinburgh Medical School: Biomedical Sciences and Euan MacDonald Centre for Motor Neuron Disease Research, The University of Edinburgh, Edinburgh, United Kingdom.; 2 Institute of Biophysics, Consiglio Nazionale delle Ricerche, Trento, Italy.; 3 UMC Utrecht Brain Center, Department of Neurology and Neurosurgery, University Medical Center Utrecht, Utrecht, Netherlands.; 4 The Roslin Institute, Royal (Dick) School of Veterinary Studies, College of Medicine and Veterinary Medicine, The University of Edinburgh, Edinburgh, United Kingdom.; 5 Centre for Systems Health and Integrated Metabolic Research, Department of Biosciences, School of Science and Technology, Nottingham Trent University, Nottingham, United Kingdom.

**Keywords:** Development, Neuroscience, Mouse models, Neuromuscular disease, Therapeutics

## Abstract

Spinal muscular atrophy (SMA) is a neuromuscular disease caused by low levels of survival motor neuron (SMN) protein. Several therapeutic approaches boosting SMN are approved for human patients, delivering remarkable improvements in lifespan and symptoms. However, emerging phenotypes, including neurodevelopmental comorbidities, are being reported in some treated patients with SMA, indicative of alterations in brain development. Here, using a mouse model of severe SMA, we revealed an underlying neurodevelopmental phenotype in SMA where prenatal SMN-dependent defects in translation drove disruptions in nonmotile primary cilia across the central nervous system (CNS). Low levels of SMN caused widespread perturbations in translation at E14.5 targeting genes associated with primary cilia. The density of primary cilia in vivo, as well as cilial length in vitro, was significantly decreased in prenatal SMA mice. Proteomic analysis revealed downstream perturbations in primary cilia-regulated signaling pathways, including Wnt signaling. Cell proliferation was concomitantly reduced in the hippocampus of SMA mice. Prenatal transplacental therapeutic intervention with SMN-restoring risdiplam rescued primary cilia defects in SMA mouse embryos. Thus, SMN protein is required for normal cellular and molecular development of primary cilia in the CNS. Early, systemic treatment with SMN-restoring therapies can successfully target neurodevelopmental comorbidities in SMA.

## Abstract 2

In utero delivery of SMN-restoring therapy corrects neurodevelopmental defects affecting primary cilia in a mouse model of spinal muscular atrophy (SMA)

## Graphical abstract

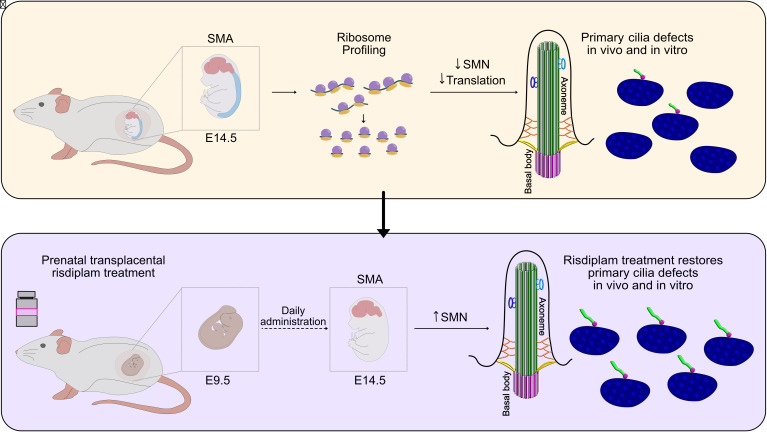

## Introduction

Spinal muscular atrophy (SMA) is an inherited neuromuscular disease with an incidence of around 1 in every 10,000 live births (1, 2). SMA is primarily characterized by progressive muscular weakness and atrophy, associated with the degeneration of spinal cord and brain stem motor neurons (3, 4). In most patients, SMA is caused by mutations in the survival motor neuron 1 gene, resulting in insufficient production of full-length, functional survival motor neuron (SMN) protein (5). The SMN protein is ubiquitously expressed in both the cytoplasm and nucleus of all cell types and is dynamically regulated during development (6, 7). High levels of SMN expression during embryogenesis undergo a significant reduction after birth, suggesting an important role for SMN during prenatal development (8). SMN has been shown to be a ribosome-associated protein, with a crucial role in translation and ribosome biology. Given its contribution to translational control of gene expression, SMN depletion leads to widespread perturbation of protein synthesis in SMA (9, 10).

In recent years, 3 disease-modifying therapies designed to increase full-length SMN protein levels have been approved to treat patients with SMA (11, 12). While these therapies represent a major breakthrough in improving the length and quality of life for patients with SMA, both clinical trial and real-world data show that they fall considerably short of a cure. Therapeutic efficacy can be enhanced by early administration, ideally before symptom onset (13–16), highlighting the critical requirement for SMN protein in presymptomatic periods preceding disease onset. As a result of these SMN-restoring treatments, individuals living with SMA now have an increased expected lifespan. Importantly, however, this has been accompanied by the emergence of new, unexpected phenotypes, including the unmasking of novel subclinical phenotypes that were not previously apparent in the pretreatment era. Among these, numerous studies have reported an emerging spectrum of neurodevelopmental comorbidities, together with cognitive and language development issues, in particular affecting children with the most severe forms of SMA (17–25). This raises fundamental questions about the role of, and requirements for, SMN protein in the developing CNS.

To explore the underpinning biology of emerging neurodevelopmental comorbidities in vivo, here we model prenatal CNS organogenesis in Taiwanese SMA mouse embryos, facilitating combined morphological and molecular analyses as well as the ability to model the impacts of therapeutic intervention. We uncovered widespread, systemic disruption of ribosome occupancy on several mRNAs throughout the CNS at embryonic day 14.5 (E14.5). Network analysis of genes with alterations in ribosome occupancy disrupted at prenatal stages in the CNS revealed involvement in processes related to primary cilia. Subsequent morphological investigation of primary cilia in the CNS in vivo and in primary neuronal cultures in vitro confirmed the presence of a primary ciliopathy in SMA. Proteomic analysis at the same time point showed multiple signaling pathways downstream of primary cilia, such as Wnt, are affected in SMA, accompanied by perturbations in cell proliferation at key stages of prenatal brain development (E14.5). Finally, we demonstrate the SMN dependence of this phenotype as well as its amenability to therapeutic intervention, by showing that it can be rescued by treatment with an SMN-restoring intervention (risdiplam) both in vitro and via prenatal transplacental delivery in vivo.

## Results

### SMN depletion leads to alterations of ribosome occupancy in the CNS of SMA mouse embryos at E14.5.

Neurodevelopmental phenotypes are a major emerging symptom in patients with SMA, particularly in severe (type I) cases after receiving an SMN-restoring therapy (17–25). This suggests a potentially important role for SMN during early (including prenatal) development of the CNS. Studying these emerging developmental phenotypes at the cellular and molecular level is virtually impossible in human patients, resulting in the need to study them in disease-relevant animal models (26). To establish whether prenatal brain development phenotypes can be reliably modeled using established mouse models of severe SMA, we initially performed Western blot analyses on brain and spinal cord across the prenatal period in the Taiwanese SMA mouse model and littermate controls. This confirmed expected, physiological levels of SMN in healthy littermate controls across the prenatal period and a clear reduction in SMA embryos (Supplemental Figure 1; supplemental material available online with this article; https://doi.org/10.1172/jci.insight.192835DS1), similar to what has previously been reported in human patients (7). Thus, SMN protein is strongly expressed throughout the mouse CNS during prenatal development, confirming that this model is suitable for studying prenatal impacts of SMN depletion in the CNS relevant to SMA.

**Figure 1:**
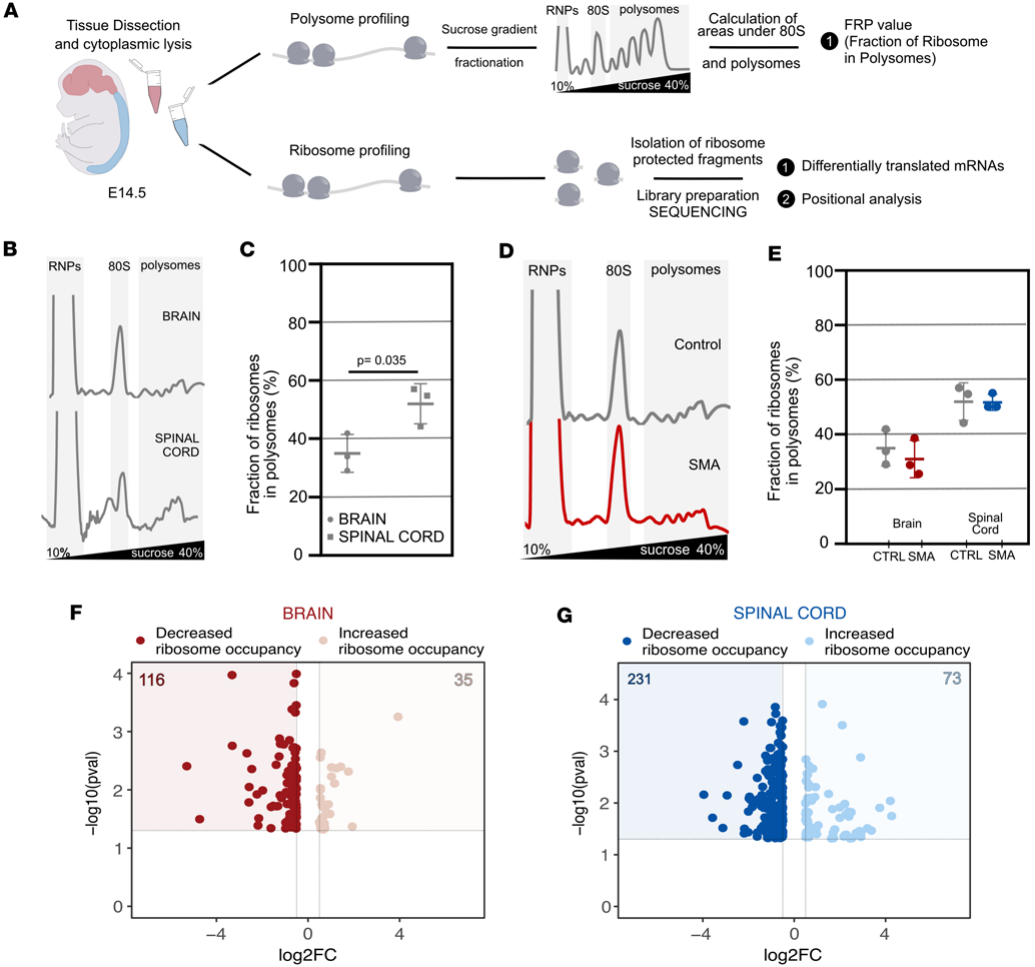
SMN depletion leads to widespread disruption in translation throughout the CNS of SMA mouse embryos. (**A**) Schematic overview of experimental design to facilitate polysome and ribosome profiling of E14.5 brain and spinal cord from control and SMA mouse embryos. RNPs, ribonucleoproteins. (**B**) Polysomal profiles of E14.5 brain and spinal cord from control mouse embryos. (**C**) FRP expressed in percentage of E14.5 brain and spinal cord from control mouse embryos. (**D**) Polysomal profiles of E14.5 brain from control and SMA mouse embryos. (**E**) FRP expressed in percentage of E14.5 brain and spinal cord from control and SMA mouse embryos. (**F** and **G**) Volcano plots showing the variations in ribosome occupancy of genes identified in brain (**F**) and spinal cord (**G**) of E14.5 controls and SMA mouse embryos. In brain, dark red dots represent DEGs with decreased ribosome occupancy, while pink dots represent increased ribosome occupancy. In spinal cord, blue dots represent DEGs with decreased ribosome occupancy, while light-blue dots represent increased ribosome occupancy. Significantly differential genes were defined by the following cutoff values: *log_2_FC*_thr = 0.5 and *pval_thr* = 0.05. *N* = 3 embryos each for control and SMA. DEGs, differentially expressed genes.

As widespread molecular changes at the proteome level have previously been reported in SMA mouse embryos (26), we next wanted to explore the mechanisms through which SMN protein may influence prenatal development of the CNS. Importantly, SMN is a ribosome-associated protein that plays a critical role in translation and ribosome biology, where SMN-primed ribosomes associating with specific mRNAs have been directly linked to SMA pathogenesis (9, 10, 27). To explore more detailed, gene-specific processes of translation during embryonic development, we performed polysome and ribosome profiling in the CNS (incorporating both brain and spinal cord) from E14.5 control and SMA Taiwanese mouse embryos (Figure 1A). Polysome profiling of cytoplasmic lysates (Figure 1B) and the estimation of the FRP from control brain and spinal cord (Figure 1C) provided a global estimation of the engagement of ribosomes on mRNAs. As a result, we observed statistically significant differences in translational efficiency in control brain compared with control spinal cord at this stage of development (Figure 1C). Notably, when we compared the estimation of the FRP between genotypes, we found no significant alterations in either the brain or the spinal cord (Figure 1, D and E).

As polysome profiles represent only an estimation of translation levels, it was not surprising that these tissues did not show a significant difference at E14.5. To obtain a genome-wide and more detailed measurement of possible alterations in ribosome engagement on mRNAs in embryonic brain and spinal cord, we performed ribosome profiling (28). This technique allowed us to specifically isolate ribosome-protected fragments and investigate the differentially translated mRNA, determining the position of the ribosomes at codon resolution. High-quality libraries, suitable for sequencing, were obtained from all animals and tissues, as illustrated by the distribution of ribosome-protected fragment lengths (Supplemental Figure 2A) and the trinucleotide periodicity along the coding sequences (Supplemental Figure 2B).

In addition to significant alterations in ribosome positioning at the beginning and end of coding sequence of mRNAs (Supplemental Figure 2B), differential analyses identified numerous transcripts with altered ribosome occupancy in both tissues (Figure 1, F and G). In brain, 116 out of 151 DEGs showed decreased ribosome occupancy (Figure 1F). Also in the spinal cord, out of 304 dysregulated genes, 231 showed decreased ribosome occupancy (Figure 1G). The overwhelming bias toward mRNAs with decreased ribosome occupancy is in line with previous observations from postnatal stages of disease and suggests that SMA pathogenesis is at least partially caused by a loss of function in translation (9, 10).

Taken together, these findings reveal that SMN is required for normal protein translation throughout the brain and spinal cord during embryonic development, with disruption occurring in advance of the (postnatal) onset of overt neuromuscular symptoms in this mouse model.

### Alterations in ribosome occupancy for genes associated with primary cilia in SMA mouse embryos.

**Figure 2:**
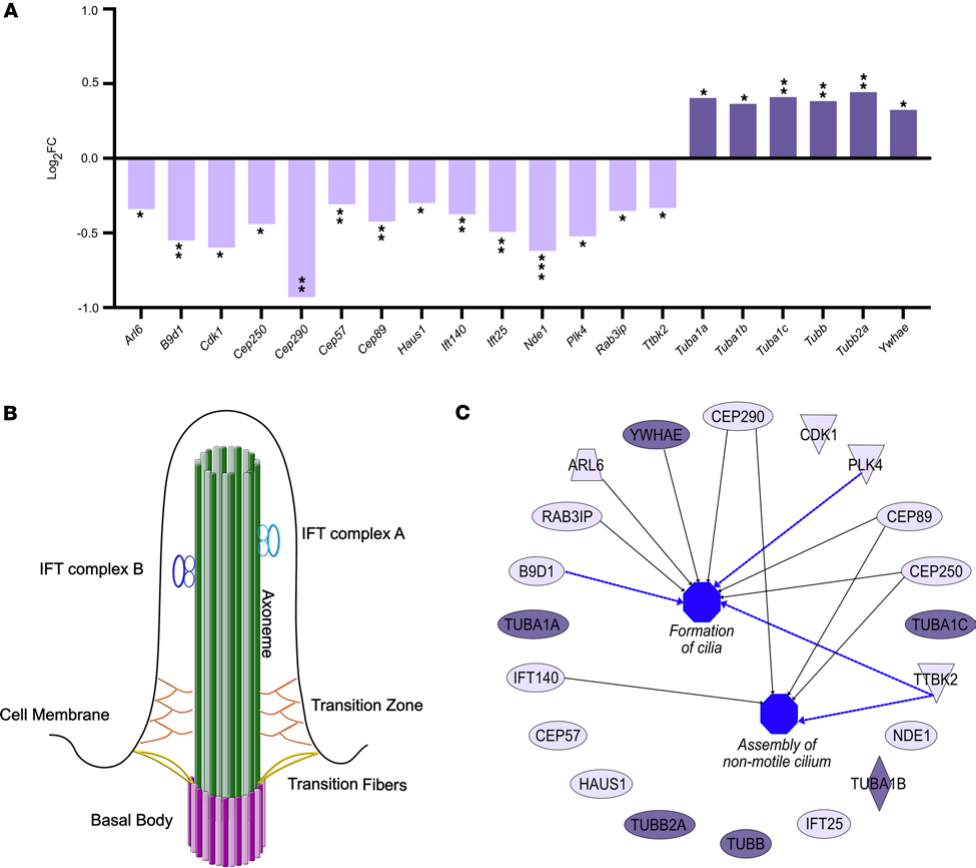
Translational defects in primary cilia genes in the CNS of SMA mouse embryos. (**A**) Bar chart showing the dysregulated genes associated to the cilium assembly canonical pathway identified by IPA. Light purple bars represent downregulated DEGs; dark purple bars represent upregulated DEGs. (**B**) Schematic of a primary cilium highlighting the main structural components: intraflagellar transport (IFT) proteins complex A and B, transition zone and fibers, basal body, and cell membrane. (**C**) Functional enrichment analysis network of cilium assembly DEGs. Downregulated molecules in light purple; upregulated molecules in dark purple. Different shapes indicate molecules with distinct biological functions. Black arrows show the enrichment of individual molecules to the functional terms formation of cilia and assembly of non-motile cilium. Blue arrows indicate a predicted inhibition state on the specific functional term. Both functional terms are highlighted in blue, indicating an overall inhibition. Statistical significance was defined using the following cutoff values: *log_2_FC*_thr = 0.3 and *pval_thr* = 0.05. *P* values were derived using the glmQLFTest function in edgeR. *P value ≤ 0.05, **P value ≤ 0.01, ***P value ≤ 0.001.

Given that depletion of SMN leads to hundreds of genes with prenatal changes in ribosome occupancy, we next wanted to specifically identify affected genes and pathways with the potential to account for developmental phenotypes observed in SMA. First, we used Ingenuity Pathway Analysis (IPA) software to identify functional clustering of the DEGs in the CNS of SMA mouse embryos, applying *log_2_FC*_thr = 0.3 and *pval_thr* = 0.05 as cutoff values (Table 1). These analyses identified a widespread disruption in several biological pathways already known to be involved in the postnatal pathogenesis of SMA, including cell cycle regulation (29, 30), cytoskeleton signaling pathway (31–33), and p53 signaling (34, 35). This suggests that molecular defects in these SMA-linked pathways can be explained by concurrent translational defects and also that they are already present prenatally. In addition to these well-characterized pathways, IPA identified changes in the translation of genes of a biologically interesting pathway not previously associated with SMA: the cilium assembly pathway. Investigation of the individual genes ascribed to this canonical pathway revealed changes in 20 genes involved in the structure and function of primary cilia, with the majority of them being downregulated (Figure 2A).

Primary cilia are nonmotile sensory organelles extending from the cell membrane, containing a microtubule-based axoneme originating from the basal body (36). The cilia cytoplasm is isolated from the cell by a specialized structure, the transition zone (TZ), which acts as a ciliary gate filtering the passage of molecules into or out of the cilium (37). The molecules that pass the TZ are carried along the axoneme by 2 intraflagellar transport complexes (IFT A and B) (38) (Figure 2B). Primary cilia have a critical role in coordinating key signaling pathways to ensure correct embryonic development, tissue homeostasis, and organ function (39). Dysfunction in these subcellular organelles leads to multisystemic disorders known as ciliopathies, and several studies have demonstrated a crucial role for primary cilia in brain development and neurodevelopmental disease, often accompanied by cognitive impairment (40–44). Several dysregulated genes identified in our dataset have been extensively described in literature and associated with well-known ciliopathies. *ARL6* and *CEP290* are 2 of the most frequently mutated genes in Bardet-Biedl syndrome, a multisystem primary ciliopathy characterized by heterogenous clinical manifestations, including cognitive impairment and developmental delay (45). Moreover, *CEP290* has also been linked to Joubert syndrome, a primary ciliopathy presenting with a distinctive midbrain-hindbrain malformation, leading to motor and cognitive impairments that manifest in early life (46, 47). Notably, mutations in *B9D1* gene are implicated in Meckel syndrome, a severe ciliopathy that is perinatally lethal due to polydactyly, kidney disease, liver fibrosis, and CNS defects (48).

Using a deeper IPA pathway enrichment of mRNAs with alteration in ribosome occupancy (see Methods), we identified a strong correlation between the identified ciliary genes and 2 independent functional terms, specifically, formation of cilia and assembly of non-motile cilium (Figure 2C). Most importantly, this in silico analysis revealed a predicted inhibition of these 2 pathways, thereby indicating primary cilia dysfunction in the CNS of SMA mouse embryos.

### Primary cilia defects in the CNS of SMA mouse embryos.

As defects in primary cilia have been associated with neurodevelopmental phenotypes similar to those reported as comorbidities in patients with SMA (21–24, 40, 42, 49–55), we next wanted to establish whether the disruption in translation of genes associated with primary cilia in SMA mouse embryos leads to corresponding morphological changes in primary cilia in vivo.

**Figure 3:**
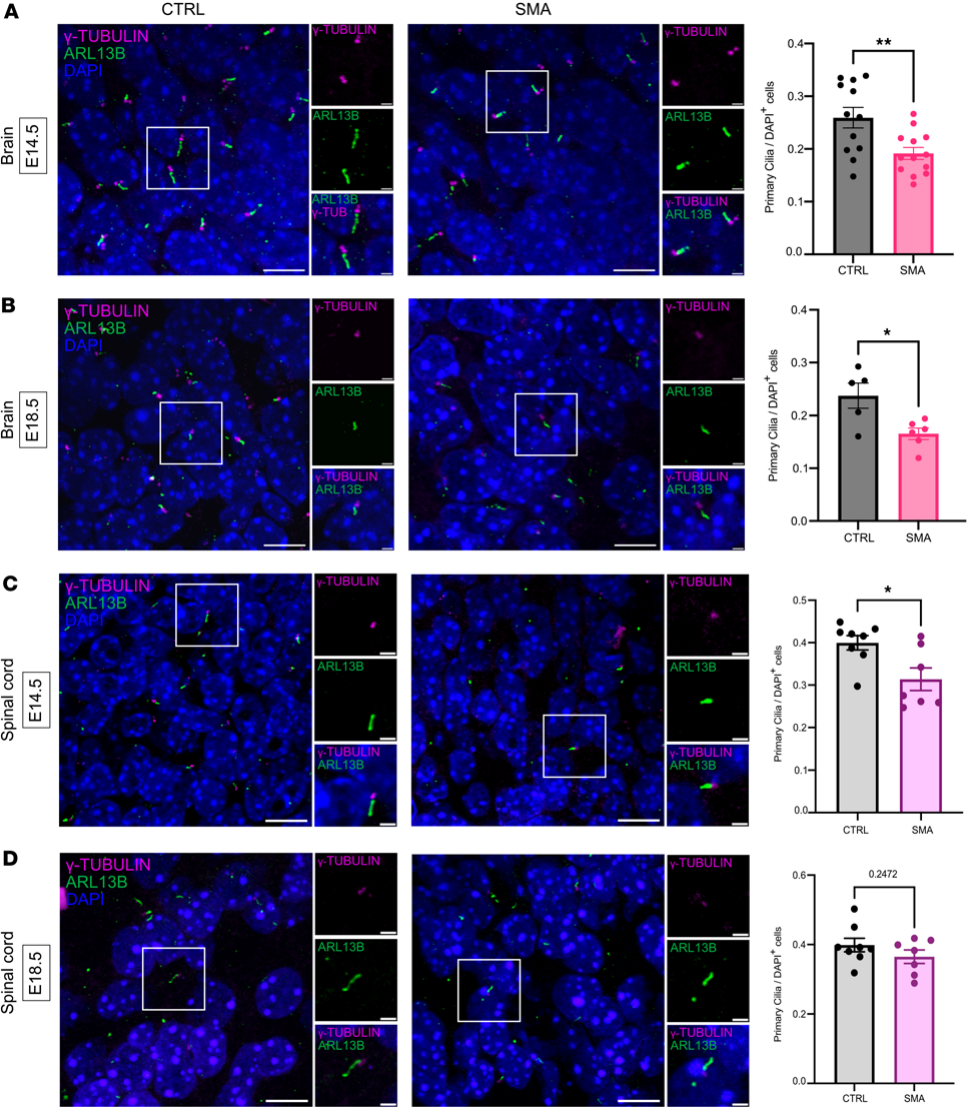
Reduced density of primary cilia in the SMA mouse embryonic hippocampus and spinal cord. (**A**) Primary cilia density quantification using ciliary markers ARL13B (axoneme, in green) and γ-TUBULIN (γ-TUB; basal body, in magenta) in the hippocampus of E14.5 and (**B**) E18.5 Taiwanese mouse embryos reveals reduced primary cilia number in SMA compared with littermate controls. Coronal paraffin sections, 10 μm thickness; scale bar: 10 μm; zoom: 2 μm. *N* = 12 embryos for control and 13 for SMA at E14.5; 5 for control and 6 for SMA at E18.5. (**C**) Primary cilia density quantification in the spinal cord of E14.5 identifies reduced primary cilia number in SMA compared with littermate controls. (**D**) No difference between genotypes observed in primary cilia density in E18.5 spinal cord. Sagittal paraffin sections, 10 μm thickness; scale bar: 10 μm; zoom: 2 μm. *N* = 8 embryos for control and 7 for SMA at E14.5 and E18.5. **P value ≤ 0.01, *P value ≤ 0.05, unpaired *t* test, scatter dot plot, mean with SEM. One data point corresponds to the average values from 3 sections per embryo.

To identify and quantify the number (density) of primary cilia in the embryonic CNS, we performed fluorescence immunohistochemistry using 2 well-established ciliary markers — ARL13B labeling the axoneme and γ-tubulin labeling the basal body — in control and SMA embryonic brain and spinal cord. Primary cilia were identifiable by the proximity of both a basal body and an axoneme. Qualitative observations in brain and spinal cord revealed the presence of primary cilia in both SMA and littermate controls and across different stages of organogenesis. Subsequent quantitative analyses showed a statistically significant decrease in the percentage of ciliated cells in SMA mouse hippocampus compared with control littermates at E14.5 (Figure 3A). To assess whether these SMA-related cilial defects persisted into later stages of embryonic brain development, we repeated the analysis in the hippocampus of E18.5 mouse embryos. A similar, statistically significant decrease in cilia density was observed in SMA mice compared with littermate controls (Figure 3B), revealing a consistent reduction in the density of primary cilia in the hippocampus of SMA mouse embryos throughout brain organogenesis. We further investigated 2 postnatal time points of postnatal (P) day 2 and P10, representing pre- and late-symptomatic stage, respectively, in the mouse model and found that cilia density was not different between SMA mice and littermate controls (Supplemental Figure 3). Thus, primary cilia disruption appears to be a largely prenatal aspect of dysregulated brain development in SMA, correlating with the higher levels of SMN protein observed at prenatal stages in both mice and humans (7).

To ascertain whether these cilial phenotypes extended into other regions of the CNS, the same quantification was performed on spinal cord sections from SMA and control embryos at E14.5 and E18.5 (Figure 3, C and D). Here, the analysis revealed a significant decrease in cilia density in the spinal cord of E14.5 SMA mouse embryos (Figure 3C), indicating that primary cilia phenotypes are conserved throughout the CNS at this crucial stage of prenatal development. However, reductions in primary cilia density did not persist to later prenatal time points (E18.5) in the spinal cord of SMA embryos (Figure 3D). The conserved manifestation of primary cilia defects at both earlier and later embryonic ages in the SMA hippocampus, but not in the spinal cord or in the hippocampus postnatally, suggests that primary cilia in brain are particularly vulnerable to low SMN levels prenatally.

### Risdiplam treatment reveals SMN-dependent morphological defects in primary cilia in primary hippocampal cell culture.

Although we identified a significantly reduced density of primary cilia in the CNS of SMA mice, changes in cilia morphology are often reported to occur alongside density changes in many ciliopathies and neurodevelopmental disorders (41, 56). To address whether core morphological features of primary cilia were also altered in SMA, we established an in vitro model system that was more suitable for quantitative morphometric analysis than brain slice preparations. This allowed us to robustly and consistently quantify cilium length, one of the most crucial morphological parameters correlated with ciliary function, which is altered in many ciliopathies (56).

**Figure 4:**
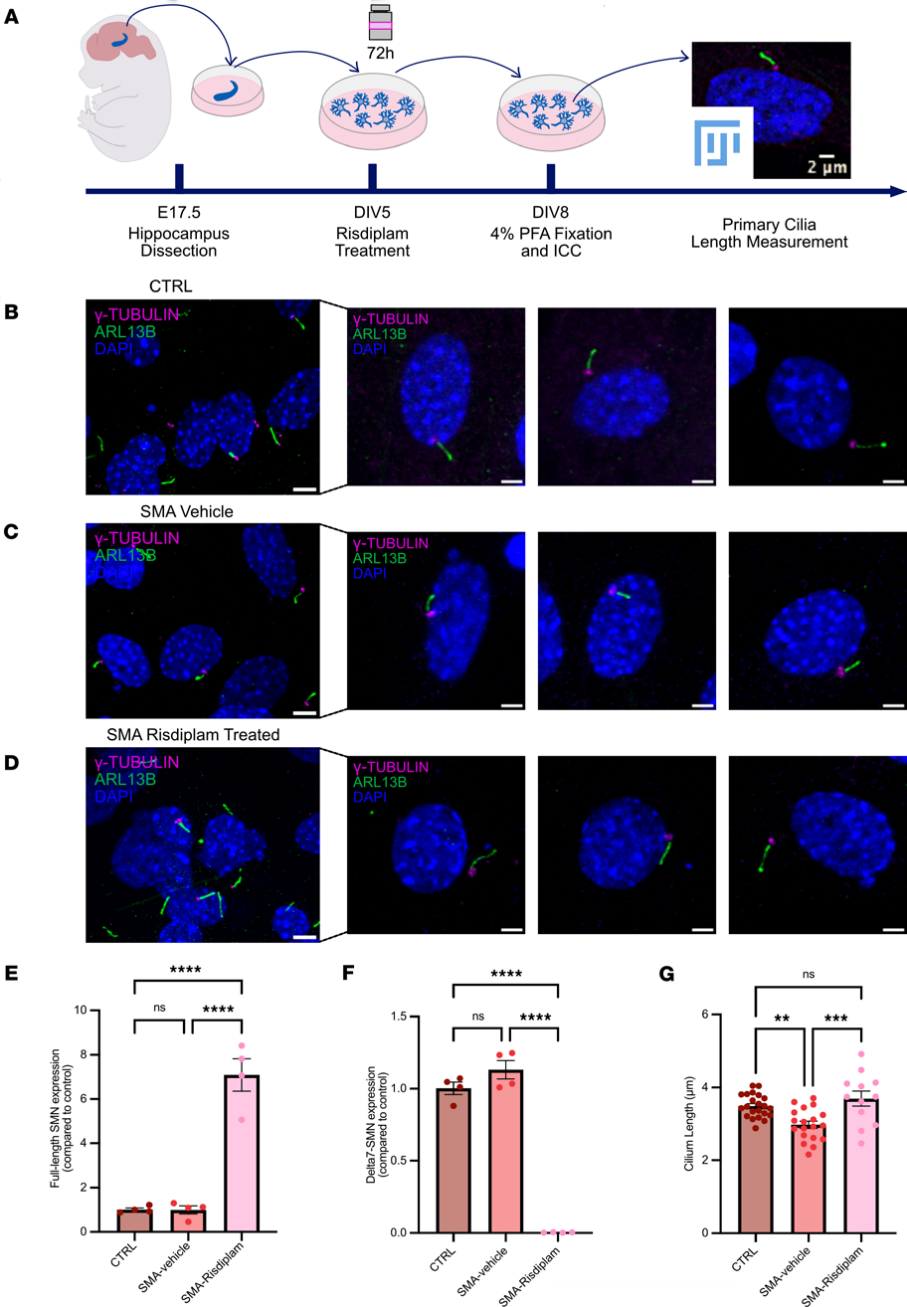
Reduced primary cilia length in SMA is restored following risdiplam treatment in primary hippocampal neurons. (**A**) Schematic showing experimental design for primary hippocampal cell culture experiments. (**B**–**D**) Representative confocal images showing primary cilia length in hippocampal cell culture from (**B**) control, (**C**) SMA vehicle-treated, and (**D**) SMA risdiplam-treated preparations. Primary cilia were labeled with the ciliary markers ARL13B (axoneme, in green) and γ-TUBULIN (basal body, in magenta). (**E**) Full-length and (**F**) *delta* *7*-*SMN* levels were quantified using real-time PCR. Bar charts show full-length *SMN* was increased, corresponding with decreased *delta 7-SMN*, in SMA hippocampal neurons treated with risdiplam. Scatter dot plot, mean with SEM. One data point corresponds to 1 embryo. *N* = 4 embryos for each group. (**G**) Primary cilia length measurement and quantification in hippocampal cell culture from control, SMA vehicle-treated, and SMA risdiplam-treated preparations. *N* = 22 embryos for control, 19 for SMA vehicle-treated, and 12 for SMA risdiplam-treated. One data point corresponds to 1 embryo. Scale bar lower magnification representative images (far left): 5 μm. Scale bar micrographs: 2 μm. **P value < 0.01, ***P value < 0.001, ****P value < 0.0001, 1-way ANOVA, scatter dot plot, mean with SEM.

We established primary neuronal cultures from the hippocampus of SMA and control mouse embryos (Figure 4A). After 8 days in vitro primary cilia were labeled with ARL13B and γ-tubulin markers. Consistent with previous observations in vivo, an initial qualitative assessment of primary cilia in vitro revealed that hippocampal cells from both control and SMA embryos possessed a clear cilium structure defined by basal body and axoneme. However, many primary cilia in SMA preparations appeared to be shorter and more truncated than those from controls. Therefore, we performed quantitative axoneme length measurements, spanning from the basal body to the tip of the cilium. This analysis revealed a significantly reduced cilium length in SMA hippocampal cell culture compared with littermate controls (Figure 4, B and C, and Supplemental Figure 4, A and B).

To assess whether cilia defects were specific to particular cell types in our primary hippocampal cell culture system, we also performed experiments costaining for NeuN (neurons) and glial fibrillary acidic protein (astrocytes). As expected, the majority of cells in our cultures were neurons (Supplemental Figure 5A), though astrocytes were also present, allowing us to compare these cell types. Quantification of ciliary length on both neurons and astrocytes revealed similar phenotypes, with shorter primary cilia axoneme in both cell types from SMA mice (Supplemental Figure 5, B and C). Thus, the SMA-linked primary cilia phenotype appears to affect multiple cell types/populations in the CNS.

Reduced density of ciliated cells in vivo and shortened cilia can also occur from the specific presence of specific basal body deficits (57). We therefore investigated the morphology of basal bodies using immunofluorescence in vitro but found no difference in gross morphology parameters of area, diameter, or perimeter (Supplemental Figure 4C). Thus, cilia defects observed in SMA are unlikely to be caused primarily by basal body deficits.

Next, we wanted to establish whether defects observed in primary cilia in SMA are amenable to therapeutic intervention by restoring SMN levels. Risdiplam, an *SMN2* splicing modifier, is 1 of 3 SMN-restoring therapies approved for use in humans with SMA and capable of increasing full-length SMN protein levels across a range of cells and tissues (58). After hippocampal dissection from SMA and control Taiwanese mouse embryos, primary neurons were plated and then treated with risdiplam for 72 hours from day 5 in vitro (DIV5). At DIV8, total RNA of hippocampal neurons was collected to analyze *SMN* expression levels using real-time PCR (59). As expected, risdiplam treatment restored full-length *SMN* levels compared with vehicle-treated SMA preparations (Figure 4, E and F).

A total of 863 primary cilia labeled with ARL13B and γ-tubulin markers to visualize primary cilia and allow accurate length measurements were analyzed from controls, along with 728 from SMA-vehicle (untreated) cells and 373 from SMA-risdiplam cells (Supplemental Figure 4D and Table 2). Strikingly, risdiplam treatment rescued primary cilia defects in the SMA cells, restoring cilia length to be indistinguishable from those observed in healthy controls (Figure 4, D and G). Thus, SMN restoration in vitro was sufficient to reverse SMA-associated primary cilia defects, verifying a critical role for SMN in regulating primary cilia during development.

### Disruption to downstream signaling pathways regulated by primary cilia in SMA.

**Figure 5:**
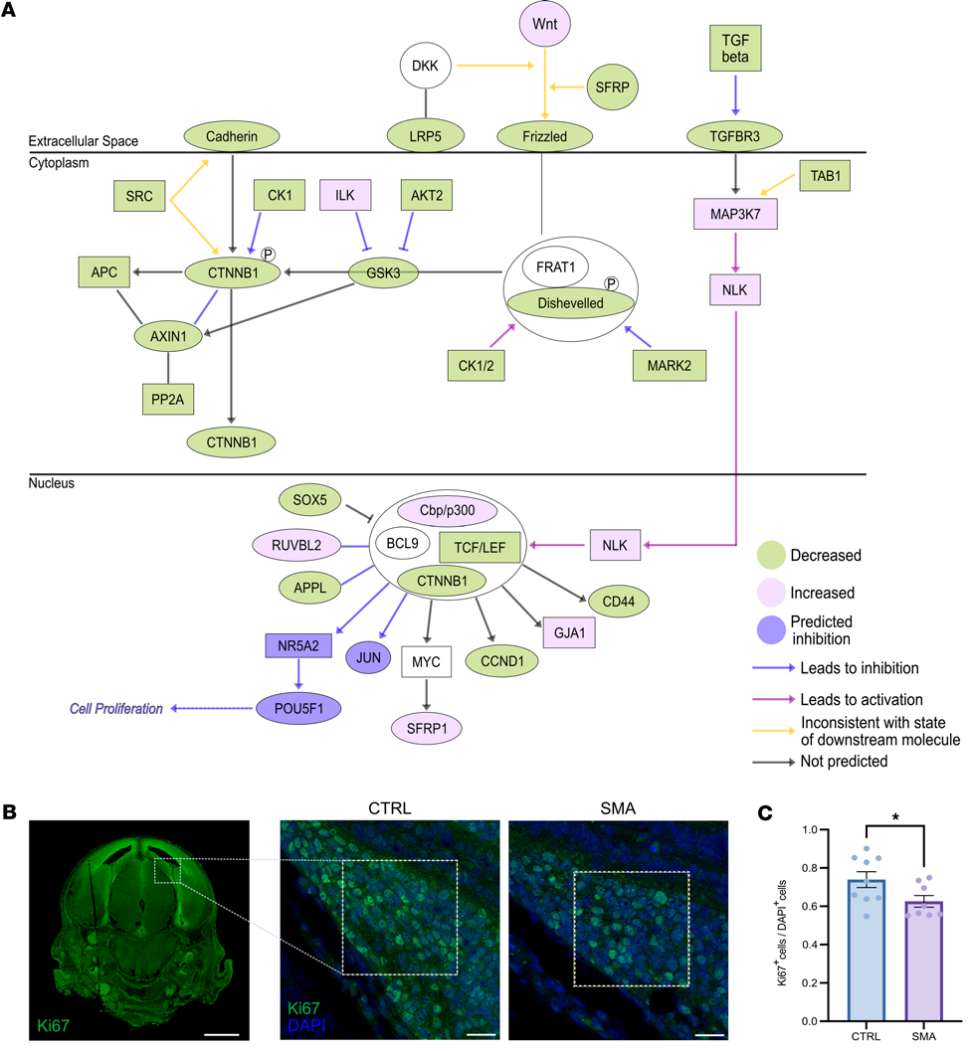
Widespread dysregulation of proteins in the Wnt signaling pathway and decreased cell proliferation in SMA embryos. (**A**) Schematic overview of the Wnt signaling pathway. Molecules highlighted in green are downregulated, in pink are upregulated, and in purple are predicted to be inhibited in SMA embryos. Purple arrows indicate inhibition; pink arrows lead to activation; yellow arrows show a discrepancy between the activation state of 1 or more molecules; and gray arrows represent an effect not predicted by IPA. (**B**) Representative coronal paraffin sections from littermate control mouse embryos at E14.5 to demonstrate whole brain topography in the mouse embryo. The relative position of the hippocampus is indicated by the white square. Cells positively labeled by the proliferative marker Ki67 are shown in green. Scale bar: 1 mm. Zoomed-in images highlight cell proliferation in the hippocampus of control and SMA mouse embryos at E14.5 shown using Ki67 labeling. Region of interest corresponding to the dentate gyrus is indicated by the white squares (39 × 39 μm). Scale bar: 10 μm. Total and proliferative cells are labeled by DAPI in blue and Ki67 in green, respectively. (**C**) Quantification of cell proliferation in the hippocampus of control and SMA Taiwanese mouse embryos reveals a significant decrease in SMA mice at E14.5. Each data point corresponds to the average values from 3 sections per embryo (*N* = 9 for control and 8 for SMA). *P value ≤ 0.05, unpaired *t* test, scatter dot plot, mean with SEM.

Having established that prenatal primary cilia phenotypes occur both in vivo and in vitro in SMA, we next investigated whether signaling pathways downstream of primary cilia were similarly affected. We therefore performed a new, targeted, in silico analysis of a recently published proteomic dataset comparing the CNS proteome in control and SMA mouse embryos at E14.5 (26, 41). IPA software analysis revealed perturbations in several signaling pathways directly linked to primary cilia (Table 3 and Supplemental Figure 6), including Wnt (Figure 5A) and Sonic Hedgehog (Supplemental Figure 6). Downregulation of both Wnt and Sonic Hedgehog pathways is known to negatively affect cell proliferation (60, 61). Consistent with this, we observed reduced cell proliferation, as indicated by Ki67 staining, in the hippocampus of SMA mice compared with littermate controls at E14.5 (Figure 5, B and C). Taken together, these findings demonstrate significant dysregulation of signaling pathways downstream of primary cilia, accompanied by a marked reduction in cell proliferation.

### In utero transplacental SMN replacement therapy increases SMN protein levels in the CNS of SMA mouse embryos and rescues primary cilia phenotypes.

Finally, given the prenatal nature of the primary ciliopathy phenotypes detailed above, we wanted to establish whether prenatal delivery of existing SMN-restoring therapeutics could intervene and correct developmental perturbations. In order to explore this, we first developed and validated an in utero transplacental therapeutic intervention using risdiplam to investigate whether SMN protein levels could be restored in SMA Taiwanese mouse embryos following treatment of the pregnant dam, with consequential impacts on the prenatal primary cilia phenotypes.

**Figure 6:**
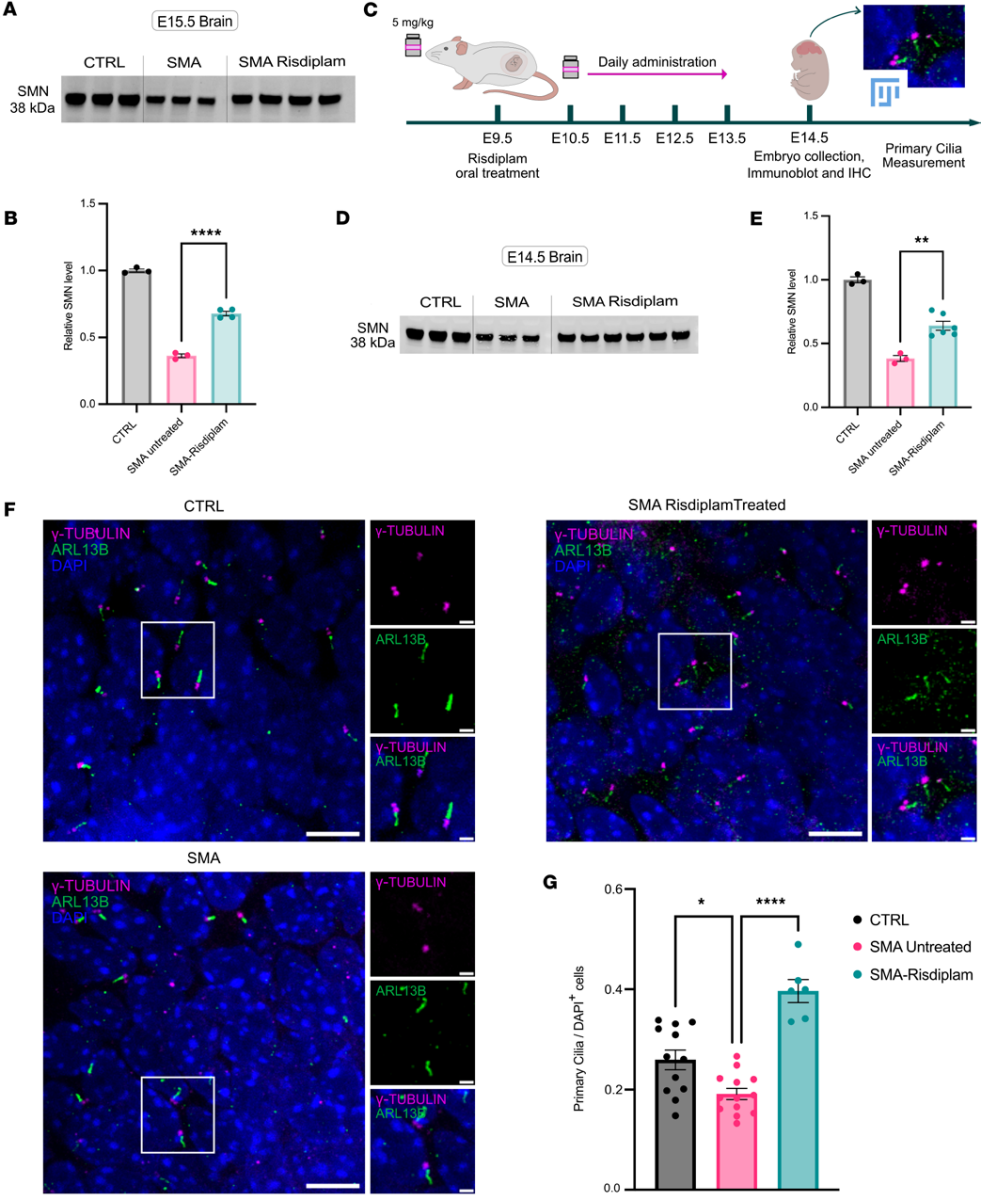
Reduced density of primary cilia in the SMA mouse embryonic hippocampus is restored following in utero risdiplam treatment. (**A**) Immunoblot of SMN levels in brain tissues from control, SMA, and risdiplam-treated SMA embryos (5 mg/kg to the dam) at E15.5. Lanes were run on the same gel but were noncontinuous (see Supplemental Figure 5A). (**B**) Quantification of relative SMN levels from **A**. *N* = 3 embryos for control and SMA, *N* = 4 embryos for risdiplam-treated SMA. (**C**) Schematic showing experimental design for short duration, in utero SMN replacement therapy via oral administration to the pregnant dam at 5 mg/kg dose. (**D**) Immunoblot of SMN levels from brain tissues from control, SMA, and risdiplam-treated SMA embryos at E14.5. (**E**) Quantification of relative SMN levels from **D**. *N* = 3 embryos for control and SMA, *N* = 6 embryos for risdiplam-treated SMA. (**F**) Representative confocal micrographs showing primary cilia in the hippocampus of E14.5 from control, SMA untreated, and SMA risdiplam-treated embryos. Primary cilia were labeled with the ciliary markers ARL13B (green) and γ-TUBULIN (magenta). Scale bar: 10 μm, zoom: 2 μm. (**G**) Quantification bar chart of **F**. Scatter dot plot with mean with SEM. One data point corresponds to 1 embryo. *N* = 12 embryos for control, 13 for SMA untreated, and 6 for SMA risdiplam treated. Data for control and untreated SMA are replotted from Figure 3A. Coronal paraffin sections, 10 μm thickness. *P value ≤ 0.05, **P value ≤ 0.01, ****P value ≤ 0.0001, 1-way ANOVA.

In an initial series of pilot experiments, 1 pregnant dam was treated with risdiplam (5 mg/kg) given by oral gavage for 5 consecutive days, starting at E10.5. The dose used in this study was adopted from doses previously established by Poirier at al., 2018 (62). Embryos were then collected at E15.5, and organs were microdissected to enable quantification of SMN protein levels. This treatment regime was sufficient to generate a 1.9-fold increase in SMN levels in the brain of SMA embryos compared with SMA embryos that did not undergo any therapeutic intervention (Figure 6, A and B, and Supplemental Figure 7A). Treatment was well tolerated by the pregnant dam, as well as the embryos, with no adverse events or phenotypes noted.

Given the success of the pilot transplacental experiments, we proceeded to perform a full set of experiments allowing comparative quantitative analyses of primary ciliopathy defects in E14.5 SMA mouse embryos. For these experiments, pregnant dams received risdiplam for 5 consecutive days via oral delivery, starting from gestational day E9.5. After daily pharmacological administration, embryos were collected at E14.5, and the brain tissue was prepared for immunoblot for SMN protein quantification and immunohistochemistry for primary cilia investigation (Figure 6C). Western blot analyses verified the prior result from pilot experiments undertaken at E15.5, revealing a significant increase in levels of SMN protein in the brain of treated SMA embryos compared with untreated embryos (Figure 6, D and E, and Supplemental Figure 7B). Strikingly, quantitative analysis of primary cilia density showed a significant increase in primary cilia number in the hippocampus of SMA embryos treated in utero with risdiplam compared with SMA-untreated embryos, restoring them to levels observed in non-SMA littermate controls (Figure 6, F and G). In addition, risdiplam treatment significantly increased levels of LRP5, one of the key Wnt signaling molecules found to be dysregulated in SMA embryos (Supplemental Figure 8). Thus, prenatal transplacental delivery of SMN-restoring therapeutics was sufficient to rescue primary ciliopathy phenotypes in SMA mice in vivo.

## Discussion

Defining the earliest changes underlying disease pathogenesis is of critical importance for uncovering the fundamental biological mechanisms driving SMA, as well as for identifying novel potential molecular targets for therapeutic intervention. Here, we demonstrate that prenatal development of the CNS is affected in SMA and identify primary cilia as contributors to SMA pathogenesis. Ribosome profiling of E14.5 brain and spinal cord revealed profound molecular defects at the translational level in the CNS of SMA mouse embryos. Importantly, pathway enrichment analysis identified genes involved in the formation and function of primary cilia. Subsequent use of both in vivo and in vitro models verified fundamental primary cilia phenotypes in SMA. Pathway analyses of proteomic datasets revealed parallel impacts on signaling pathways downstream of primary cilia in SMA. Importantly, these defects were shown to be SMN dependent and were rescued following therapeutic intervention with risdiplam, including via prenatal transplacental delivery of SMN-restoring therapeutics in vivo.

Primary cilia are microtubule-based structures protruding from the surface of almost all eukaryotic cells. As sensory organelles, primary cilia are characterized by a specialized structure in which each component is essential for the correct functioning of the cilium (41). Among those, the TZ plays a crucial role in controlling the entry and exit of cargo proteins essential for cilia assembly and signaling (37). Here, we found that several genes that encode TZ components, such as *Arl6*, *B9d1*, and *Cep290*, have decreased ribosome occupancy in the CNS of SMA mouse embryos. Alongside the TZ, IFT proteins are also essential for the correct assembly and maintenance of cilia, as they mediate the bidirectional transport of structural and signaling molecules along the microtubular axoneme (63). *Ift140* and *Ift25* genes encode 2 crucial components of the IFT system that were also dysregulated in the CNS of SMA mouse embryos. Importantly, IFT25 facilitates anterograde transport to the ciliary tip of smoothened and glioma-associated oncogene, 2 proteins essential for the activation of the Sonic Hedgehog signaling pathways, a key modulator of embryonic development and tissue homeostasis (43, 64, 65). Thus, SMN depletion appears to impact the translation of a wide range of primary cilia genes likely to result in significant developmental perturbations in these key structures. Further studies exploring the precise molecular regulation of cilia formation and function in the context of SMA are therefore warranted.

Notably, in our in vitro experiments, we observed the presence of cilia phenotypes in both neuronal cells and astrocytes. Further work will be required to establish a full, cell type–specific analysis of cilia phenotypes across the CNS in SMA, but the current data suggest that dysregulation is likely to be widespread.

One particularly interesting observation from the current study was that the magnitude of primary cilia varied across CNS regions and developmental time points: There was a reduction in the density of ciliated cells in the hippocampus at embryonic days E14.5 and E18.5, but not postnatally, and prenatal reduction occurred only at E14.5 in the spinal cord. This is likely best explained by differing developmental timelines of these structures. The spinal cord undergoes region patterning and cell identity establishment as early as E9.5 in mice, with the transition from neural progenitor cells to postmitotic neurons largely complete embryonically (66). In contrast, in the hippocampus, although region specification signals appear by E10.5, its maturation extends to postnatal ages in mice (67, 68).

Acting as signaling hubs, primary cilia have a crucial role during embryogenesis, detecting and transducing extracellular signals that coordinate fundamental biological processes, including cell cycle progression and cell proliferation (41). For this reason, primary cilia dysfunction leads to a spectrum of severe prenatal and developmental diseases, known as ciliopathies. The clinical manifestations of ciliopathies are multisystemic and include brain malformations, cognitive impairment, cardiac defects, renal malformations, liver disease and musculoskeletal abnormalities, as described in well-characterized ciliopathies such as Joubert, Meckel-Gruber, and Bardet Biedl syndromes (46–49, 69–72). Furthermore, primary cilia defects have already been associated with other neurodegenerative and neuromuscular disorders, including amyotrophic lateral sclerosis (40, 73–75). Thus, the prenatal primary cilia defects reported here would be predicted to contribute directly to neurodevelopmental symptoms being reported in patients with SMA.

From a clinical viewpoint, our work is potentially relevant for better understanding of cognitive impairments in patients with SMA treated with currently available therapies. Numerous recent patient-based studies have reported deficits in attention, memory, and language, as well as autism-like symptoms, such as difficulties in social communication and interaction, in SMA patients with and without SMN-restoring therapies (17, 19–25). The hippocampus is known to play a key role in memory and cognition in humans (76), with previous studies showing that depletion of primary cilia leads to hippocampus-dependent learning and memory deficits in mice (50, 54). Moreover, MRI studies have revealed hippocampal dysgenesis and volume loss in patients with Bardet Biedl syndrome, highlighting the impact of primary cilia in cognition (49, 77). Taken alongside the current findings of defects in this brain region during embryonic development in SMA mice, monitoring of developmental processes in key brain regions such as the hippocampus is likely to be required for patients with SMA. This highlights the need for routine assessment of neurocognitive abilities in patients with SMA (including those receiving SMN-restoring treatments), as well as the importance of consistent neonatal, or perhaps even prenatal, screening for SMA. Such monitoring of patients with SMA and their clinical manifestations will also be key for the understanding the heterogeneity of phenotypes that exists among patients. For example, cognitive impairment and intellectual disability have been observed less frequently in patients with milder forms of SMA (type II and III) (78, 79), in contrast with pediatric patients living with the most severe form of SMA (type I) (17, 18, 21).

There is now unquestionable preclinical and clinical evidence demonstrating that earlier (ideally presymptomatic) intervention in SMA provides better therapeutic outcomes and enhances quality of life for patients and their families. To date, several studies have performed in utero SMN replacement using antisense oligonucleotides, viral vectors, or small molecules in preclinical mouse models (80–83), demonstrating benefits on neuromuscular aspects of the disease (16). The findings of the current study reinforce the need for early treatment, suggesting that prenatal changes occurring during CNS development need to be targeted to address neurodevelopmental comorbidities in SMA while providing important proof of principle that transplacental in utero SMN replacement can reverse neurodevelopmental SMA phenotypes. Importantly, these preclinical findings have recently been shown to have significant potential for translation into the patient context because of a publication detailing the first case of prenatal therapy in humans, where risdiplam was administered orally to a mother during gestation, demonstrating the efficacy of transplacental drug delivery and potential early intervention benefits (84). Strikingly, the dosing regimen used for prenatal transplacental treatment in the current study revealed a substantial impact on primary cilia even after a short duration of treatment (a maximum of 5 days out of a total gestation period lasting approximately 21 days in mice). Taken together, these findings suggest that relatively short prenatal treatment with SMN-restoring therapeutics during critical time periods of brain development is likely to be sufficient to ameliorate neurodevelopmental aspects of SMA.

## Methods

### Sex as a biological variable.

Our study examined both sexes that were determined by PCR amplification of X chromosome genes with divergent Y chromosome gametologs (85). Although both sexes were used throughout the entire study, sex was not considered as a biological variable.

### Statistics.

All results are expressed as mean and SEM from at least 5 independent samples per group. For each embryo, 3 consecutive brain sections were analyzed and averaged together. Differences between the 2 genotype groups (control and SMA) were analyzed by a 1- or 2-tailed unpaired parametric *t* test. Differences among 3 groups (control, SMA vehicle, SMA risdiplam treated) were analyzed by ordinary 1-way ANOVA using Tukey’s correction for multiple comparisons. All statistical analyses were performed using GraphPad Prism 10. A *P* value less than 0.05 was considered significant.

### Study approval.

All animals were bred and handled following the UK Animals (Scientific Procedures) Act, 1986. Procedures were approved by the internal ethics committee at the University of Edinburgh and following UK Home Office regulations (Project Licence number: PPL PP1567597).

### Data availability.

All data values for all graphs, and values behind any reported means in the manuscript or supplement, are reported and accessible in the Supporting Data Values XLS file. Raw proteomic data used in Figure 5 are available via Edinburgh DataShare: https://doi.org/10.7488/ds/2776 Ribosome profiling data have been deposited in the National Center for Biotechnology Information Gene Expression Omnibus (accession code GSE295681). Dysregulated gene list from ribosome profiling is available as a source data file, Supplemental Data 1. Detailed methods can be found in Supplemental Methods.

Any other information not already included in the manuscript but used in the analysis is available to any researcher for purposes of reproducing or extending the analysis upon contacting the corresponding author.

## Author contributions

The order of co–first authors and co–senior authors was determined based on their respective and equal contributions. The study was conceptualized by FG, YTH, AALM, EJNG, GV, and THG. Polysome and ribosome profiling experiments were conducted by FG, GS, IS, DD, EJNG, and GV, and the resulting data were analyzed by M Paganin and GV. NCHL, M Pronot, and MAC prepared primary hippocampal cell cultures, and KJM helped with their maintenance and RNA extraction. RAK and TMW provided support for IPAs. FG performed all primary cilia experiments, including tissue and cell collection, staining, imaging, and analysis. YTH carried out transplacental in utero treatment and SMN protein and RNA quantification. The methodology of the study was developed by FG, YTH, AALM, GS, IS, HC, KMEF, TMW, RAK, EJNG, GV, and THG, with the supervision of TMW, EJNG, MAC, GV, and THG. Funding acquisition was carried out by MAC, TMW, EJNG, GV, and THG. The original draft was written by FG, YTH, AALM, and THG, while all authors participated in the review and editing of this manuscript.

## Funding support

European Union’s Horizon 2020 research and innovation program (project SMABEYOND, No. 956185) (FG, GS, EJNG, GV, and THG).

SMA Europe (project grant funding) (AALM, EJNG, and THG).

My Name’5 Doddie Foundation (project grant funding) (YTH, HC, and THG).

Telethon Italy (GGP19115 and GMR23T1048) (GV).

European Union within the Ministry of Education and Research National Recovery and Resilience Plan National Center for Gene Therapy and Drugs based on RNA Technology (Project no. CN00000041 CN3 RNA) (GV).

Caritro Foundation (Rif.Int. 2021.0571) (GV).

Epilepsy Research Institute (Project No. 2003) (MAC).

The Wellcome Trust (Project No. 204954/Z/16/Z) (MAC).

European Union Marie Skłodowska-Curie Fellowship, UK Research & Innovation Guarantee (Project No. EP/Y024559/1) (MAC).

LifeArc (Pathfinder Award) (HC and THG).

Academy of Medical Sciences Starter Grant for Clinical Lecturers (KMEF).

Medical Research Council Clinician Scientist Fellowship/Motor Neuron Disease Association Lady Edith Wolfson Clinical Fellowship (KJM & KMEF).

TMW & RAK acknowledge strategic investment from the Biotechnology and Biological Sciences Research Council, including Institute Strategic Program Grant funding (reference BB/X010945/1).

## Supplementary Material

Supplemental data

Supplemental data set 1

Unedited blot and gel images

Supporting data values

## Figures and Tables

**Table 1:**
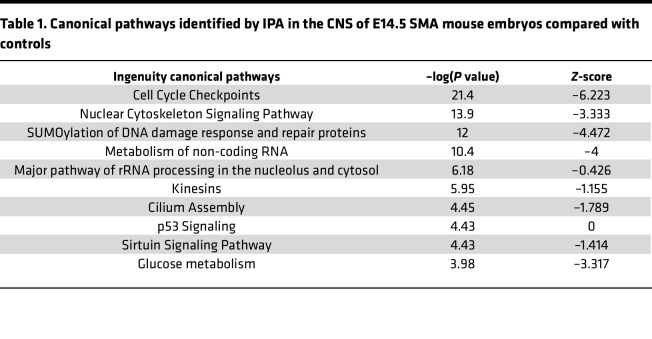
Canonical pathways identified by IPA in the CNS of E14.5 SMA mouse embryos compared with controls

**Table 2:**
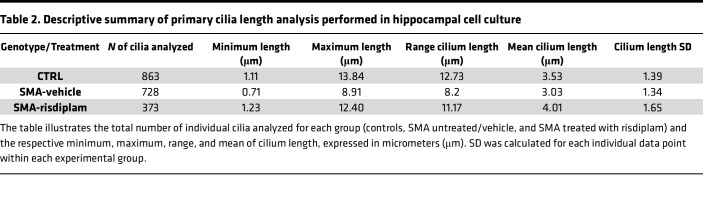
Descriptive summary of primary cilia length analysis performed in hippocampal cell culture

**Table 3:**
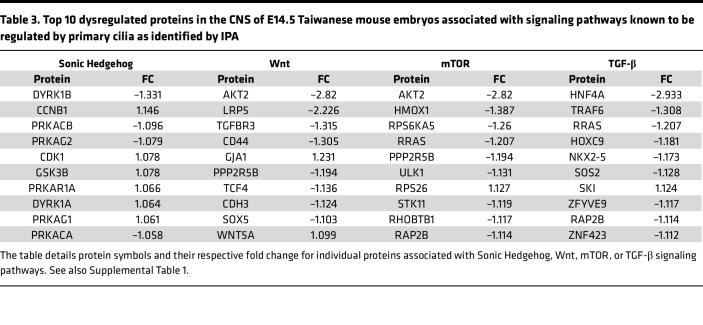
Top 10 dysregulated proteins in the CNS of E14.5 Taiwanese mouse embryos associated with signaling pathways known to be regulated by primary cilia as identified by IPA

## Version 1 09/09/2025

In-Press Preview

## Version 2 10/22/2025

Electronic publication
