# Supplementary material for: Prenatal SMN-dependent defects in translation uncover reversible primary cilia phenotypes in spinal muscular atrophy
Source: JCI Insight. 2025 Sep 9;10(20):e192835. doi: 10.1172/jci.insight.192835 (PMC12581668; doi:10.1172/jci.insight.192835)
Supplement: Supplemental data [file jciinsight-10-192835-s141.pdf]

Prenatal SMN-dependent defects in translation uncover reversible primary cilia phenotypes in spinal muscular atrophy

Federica Genovese[1$], Yu-Ting Huang[1$], Anna A.L. Motyl[1], Martina Paganin[2], Gaurav Sharma[2], Ilaria Signoria[3], Deborah Donzel[2], Nicole C.H. Lai[1], Marie Pronot[1], Rachel A. Kline[4], Helena Chaytow[1], Kimberley J. Morris[1], Kiterie M.E. Faller[1], Thomas M. Wishart[4,5], Ewout J.N. Groen[3], Michael A. Cousin[1], Gabriella Viero[2^], Thomas H. Gillingwater[1*^]

[1]Edinburgh Medical School: Biomedical Sciences & Euan MacDonald Centre for Motor Neuron Disease Research, The University of Edinburgh; Edinburgh, UK.

[2]Institute of Biophysics, CNR Unit; Trento, Italy.

[3]UMC Utrecht Brain Center, Department of Neurology and Neurosurgery, University Medical Center Utrecht, Utrecht, Netherlands.

[4]The Roslin Institute, Royal (Dick) School of Veterinary Studies, College of Medicine and Veterinary Medicine, The University of Edinburgh; Edinburgh, UK.

[5]Centre for Systems Health and Integrated Metabolic Research, Department of Biosciences, School of Science and Technology Nottingham Trent University, Nottingham, UK

*Corresponding author t.gillingwater@ed.ac.uk Tel: +44 (0)1316503724

Edinburgh Medical School: Old Medical School, Doorway 3, Teviot Place, Edinburgh

[$] These authors are co-first authors and contributed equally to this work

^ These authors are co-senior authors and contributed equally to this work

Competing interests:

THG has provided advisory services concerning SMA for Roche and Novartis.

Supplemental material: methods

Study design. This study was designed to investigate the systemic developmental defects of Spinal Muscular Atrophy in the Taiwanese mouse model, with a particular focus on translation. Exploring brain development throughout the entire period of mid-late organogenesis and performing ribosome profiling, we unveiled primary cilia as a molecular mechanism underlying embryonic SMA pathogenesis. Sample size was determined by preliminary data and power calculations. All data were quantified and assessed blinded to genotype/treatment. Both genders were included in this study and determined by PCR amplification of X chromosome genes with divergent Y chromosome gametologs (1). Although both genders were used throughout the entire study, sex was not considered as a biological variable.

Animals. The Taiwanese model of SMA (2, 3), was maintained as previously described, having been originally obtained from Jackson Laboratories (strain 005058). We refer to *Smn+/−; SMN2tg/0* embryos as healthy controls, and *Smn−/−; SMN2tg/0* as SMA embryos. The morning of vaginal plug discovery was counted as embryonic day 0.5 (E0.5), and the embryos were collected when they reached the age of interest. For an up-to-date overview of the benefits and limitations of this mouse model please see the recent review by Signoria and colleagues (4).

Pregnant dams were sacrificed at the desired time point and then embryos were collected individually. Each embryo was weighed, and the stage of development was assessed accordingly (5). Heads and bodies of embryos or postnasal brains were separated prior fixation and tail tips were used for genotyping and sex determination.

For in utero SMN replacement therapy experiments, four pregnant dams were administered risdiplam at 5 mg/kg for 5 consecutive days by oral gavage (PO) prior the tissue collection at

E14.5 or E15.5. Risdiplam was dissolved in 0.5% Hydroxypropylmethylcellulose, 0.1% Tween-80 (6).

Immunohistochemistry.

Microtomy. Whole embryos' heads and bodies were fixed in 4% paraformaldehyde overnight at 4°C. On the next day, tissues were rinsed with PBS and stored in 70% ethanol prior paraffin-embedding. Samples were dehydrated using an automated tissue processor and then infiltrated with histological wax. 10μm-thick coronal (heads or brains) and sagittal (bodies) sections using microtome were collected on SuperfrostTMPlus slides and kept overnight at 37°C.

Fluorescence immunohistochemistry. Wax sections were deparaffinised and rehydrated with Xylene and ethanol, respectively. Antigen retrieval was performed by maintaining the slides at a sub-boiling temperature for 20 minutes in Tris-EDTA or sodium citrate buffer depending on the primary antibodies. (10 mM Tris-base and 1mM EDTA solution pH 9.0 for ARL13B and γ-TUBULIN; 10 mM sodium citrate buffer, pH 6 for Ki67). Slides were then left to cool down at room temperature for 30 minutes.

The sections were washed two times in 0.1% Triton X-100 in TBS and immersed in corresponding blocking solution at room temperature for one hour (10% donkey serum in TBS for anti-ARL13B and anti-γ-TUBULIN; 1% BSA, 10% donkey serum in TBS for Ki67).

The sections were then incubated with primary antibodies overnight at 4°C (anti-ARL13B and anti-γ-TUBULIN were diluted in 5% donkey serum in TBS; anti-Ki67 was diluted in 1% BSA, 10% donkey serum in TBS). Primary antibodies used in this experiment were: rabbit polyclonal anti-Ki67 antibody (Abcam #ab15580 1:200); rabbit polyclonal anti-ARL13B antibody (ProteIntech #17711-1-AP, 1:200); and mouse monoclonal anti-γ-TUBULIN antibody (Sigma #T6557 mouse mAb, 1:500).

After three washes of 10 minutes each in 0.025% Triton X-100 in TBS, sections were then incubated with secondary antibody at room temperature for two hours (1:400 donkey anti-rabbit Alexa Fluor 488, #A-32790, for Ki67 and ARL13B; 1:400 donkey anti-mouse Alexa Fluor 594, A-21203, for γ-TUBULIN). After three washes of 10 minutes each in 0.025% Triton X-100 in 1xTBS, slides were counterstained by 4',6-diamidino-2-phenylindole (DAPI) and mounted with Mowiol.

Quantitative western blotting. Tissue collection and western blot procedures have been previously reported (7). In brief, tissues were snap-frozen in dry ice and stored at -80 °C freezer until use. Tissues were homogenized in radioimmunoprecipitation assay buffer (ThermoFisher Scientific) with Halt™ protease inhibitor (#78429, ThermoFisher Scientific). Protein concentrations were determined using the bicinchoninic acid method. 10 or 30 μg of total protein was used in longitudinal SMN expression experiment or in in utero SMN replacement experiment, respectively. After electrophoresis, proteins were transferred to a polyvinylidene difluoride membrane (iBlot2, ThermoFisher Scientific). Total protein was quantified by Revert™ Total Protein Stain (520nm or 700nm, LI-COR). Membranes were then blocked in Odyssey PBS blocking buffer (LI-COR) or EveryBlot blocking buffer (Bio-Rad), followed by incubation anti-SMN antibody solution (BD Bioscience #610646, 1:1500) or anti-LRP5 antibody (Cell Signaling #5731, 1:1000) overnight at 4°C. The next day, membranes were washed with 1× PBS at RT before and after and secondary antibody incubation (LI-COR). Membrane is dried followed by image acquisition on an LI-COR Scanner Odyssey M.

To allow longitudinal comparison of SMN levels across ages, we employed the internal control samples, accounting for variability across different blots (7). Internal control samples were mixture of control brain tissues from E16.5 and each gel contained triplicate of internal controls. Comparisons were performed using mixed-effect linear models (7, 8).

Primary culture of mouse embryonic hippocampal neurons.

Ex vivo hippocampal neuron preparation. Primary hippocampal cell cultures were prepared as previously described (9). Briefly, hippocampal tissues from E17.5 Taiwanese mice embryos were dissociated with papain (Worthington Biochemical PAP2, reconstitute to 10 U/mL in PBS) and placed in a 37°C waterbath for 20 minutes. Supplemented DMEM/F12 (with penicillin/streptomycin solution and 5% foetal bovine serum (Life Technologies #21331-020) was then added to the dissociated tissues to a final volume of 5 mL followed by centrifuging at 1500 rpm for 5 minutes at room temperature. The pellet containing hippocampal cells was resuspended in pre-warmed supplemented Neurobasal (with 1x B-27 supplement, 0.5 mM L-glutamine and 100 U/mL penicillin–100 µg/mL streptomycin) media. Each embryo's hippocampus was individually dissociated and plated on individual 6-well plates. $6x10^4$ cells per well were plated onto poly-D-lysine coated coverslips in a 6-well plate. The cells were allowed to adhere in a 37°C/5% CO_2 incubator for one hour. Supplemented Neurobasal (2 mL) was then added to each well and cells further incubated at 37°C/5% CO_2. Two days later, Cytosine beta-D-arabinofuranoside (Ara-C) was added to the culture at a final concentration of 1 µM per well to prevent glial proliferation.

Pharmacological treatment with risdiplam. Hippocampal neurons were treated for 72h from day in vitro 5 (DIV5). For each 6-well plate, half (3 wells) were treated with risdiplam (Cayman Chemical #29028), while the other half received the same quantity of sterile deionised water as control. Risdiplam dissolved in deionised water was used at final concentration of 0.5 µM. Treatment was replaced every 24h.

SMN mRNA levels following treatment with risdiplam. Total RNA was extracted from cultured hippocampal cells using a RNeasy Plus Kit (Qiagen). Total RNA (80 ng) was used for

first strand cDNA synthesis, using a Maxima First Strand cDNA Synthesis Kit for RT-qPCR (Thermo). Quantitative real-time PCR was performed on CFX Opus Deepwell system (Bio-Rad) using PowerUp™ SYBR™ Green Master Mix for qPCR (Thermo) according to the manufacturer's instruction. Human-specific full-length *SMN2* primers (Forward, 5'-ATA CTG GCT ATT ATA TGG GTT TT-3'; Reverse, 5'-TCC AGA TCT GTC TGA TCG TTT C-3' [133 bp]), human-specific *SMN delta7* primers (Forward, 5'-TGG ACC ACC AAT AAT TCC CC-3'; Reverse, 5'-ATG CCA GCA TTT CCA TAT AAT AGC C-3' [125 bp]) and mouse-specific *Gapdh* primers (Forward, 5'-AAT GTG TCC GTC GTG GAT CTG A-3'; Reverse, 5'- GAT GCC TGC TTC ACC ACC TTC T -3' [83 bp]) were used (10, 11).

The PCR program was as follows:

50°C 2 min,

95°C 10 seconds] 40 cycles

60°C for 30 seconds

5 seconds each at 0.5°C increments between 65°C and 95°C for melt curve analysis

The cycle at which the amount of fluorescence was above the threshold (Ct) was detected. Relative full length and *delta 7 SMN* expression for each sample was firstly normalised to its *Gapdh* level then compared to controls.

Fluorescence immunocytochemistry. After 8 days in vitro (DIV8), cells were fixed in 4% paraformaldehyde for 15 minutes. The coverslips were treated in 0.1% Triton X-100 in TBS for permeabilization for 20 minutes and immersed in blocking solution (20% donkey serum in TBS) at room temperature for one hour. The coverslips were then incubated with primary antibodies overnight at 4ºC (anti-ARL13B 1:500; anti-γ-TUBULIN 1:2000; anti-GFAP ThermoFisher #13-0300 1:200; anti-NeuN Merck #MAB377 1:100, in 10% donkey serum in TBS).

After three washes of 10 minutes each in 0.025% Triton X-100 in TBS, coverslips were incubated with secondary antibody at room temperature for two hours. After three washes of 10 minutes each in 0.025% Triton X-100 in TBS, the coverslips were counterstained by DAPI then mounted with Mowiol.

Microscopy. Immunofluorescent whole brain sections from control embryos stained with the proliferation marker Ki67 were imaged on LI-COR Scanner Odyssey M using 5 resolution and 2.50 mm focus offset.

Immunofluorescent sections were imaged on an inverted confocal microscope (Nikon A1R). For Ki67 and DAPI staining, were imaged using a 40x magnification oil-immersion lens and z-stacks were acquired with Galvano scanning at 1 μm step. For ARL13B, γ-TUBULIN and DAPI staining used for primary cilia investigation, three consecutive sections per mouse were imaged using a 60x magnification oil-immersion lens. Z-stacks were acquired with Galvano scanning at 0.3 μm step. The precise sections used for the analysis were selected by the identifications of neuroanatomical landmarks illustrated in the Schambra Prenatal Mouse Brain Atlas. Cilia quantification was performed by collapsing five z-stacks in a max intensity z-projection on ImageJ. The field of view was divided in three 60x60 μm squares, randomly distributed around the anatomical region of interest. DAPI and ciliated cells quantification was performed in each of the squares and then averaged.

Immunofluorescent hippocampal cell cultures stained for ARL13B, γ-TUBULIN, NeuN, GFAP and DAPI were imaged on an inverted confocal microscope (Nikon A1R) using a 60x magnification oil-immersion lens and z-stacks were acquired with Galvano scanning at 0.3 μm step. Cilia morphological measurements were performed on the entire field of view (208x208 μm) by collapsing all z-stacks in a max intensity z-projection on ImageJ.

Imaris Software was used for 3D rendering and visualisation of hippocampal primary cilia from representative control and SMA embryos.

Translatomics.

Polysome profiling. For sucrose gradient preparation, solutions were prepared using a specific buffer composition (10 mM Tris-HCl pH 7.5, 10 mM MgCl2, 10 mM NaCl) with varying sucrose concentrations. Small gradients (4 mL) were prepared by overlaying 1.6 mL of 40% (w/v) sucrose buffer and filling the tube with 10% (w/v) sucrose buffer. The gradient was formed by keeping the tube horizontally for 120 minutes at 4°C.

For cytoplasmic lysates preparation, mouse-frozen tissues were used following the protocol described in 2017 study (12). Tissues were pulverized in liquid nitrogen using a pestle and a mortar. After pulverization, 400-800 µL of tissue polysome lysis buffer (12) was used for powder resuspension. Lysates were kept for 17-20 minutes on ice for cell lysis. Then samples were centrifuged twice at 12,000 rpm in Eppendorf Centrifuge 5417 for 10 min and the supernatant was collected. Samples were loaded on 4 mL polyallomer ultracentrifuge tubes (Beckman) containing 10%-40% (w/v) linear sucrose gradient and ultracentrifuged for 90 min at 40,000 rpm at 4 °C in Beckman Optima XPN-100 Ultracentrifuge in SW41 rotor. Sucrose fractions were collected using Teledyne Isco model 160 gradient analyzer equipped with a UA-6 UV/VIS detector to measure the absorbance at 254 nm. Plotting absorbance vs fraction number yields a polysome profile.

Fraction of ribosomes in polysomes. The fraction of ribosomes in polysomes (FRP) is measured based on the polysome profiling curve as follows:

$$\%FRP = \frac{\text{Area under the polysomes}}{(\text{Area under the 80S} + \text{Area under the polysomes})} \times 100$$

Ribosome profiling and library preparation. Brain and spinal cord were collected from control and SMA embryos at E14.5, snap frozen and then stored at -80°C until used.

Cytoplasmic lysates were prepared as described previously(12). Briefly, the tissues were pulverised with a sterile pestle and mortar in liquid nitrogen, and then 400-800 μL of tissue was added to ribosome lysis buffer (10mM Tris-HCl pH 7.5, 10mM $MgCl_2$, 10 mM NaCl, 1% w/V TritonX-100, 5 U/mL DNAseI, 800 U/mL RiboLock RNAse Inhibitor, 1 mM DTT, 200 μg/mL cycloheximide, 1% w/v Na-deoxycholate). To facilitate cell lysis, lysates were incubated on ice for 17-20 minutes followed by centrifuging twice at 12,000 rpm for 10 min at 4°C to remove tissue debris, mitochondria, and nuclei. Supernatants was measured for the 260nm absorbance, the final NaCl salt concentration of the lysate was adjusted to a final salt concentration of 100 mM.

Ribosome purification was achieved by treating lysates with 10 U/Abs RNAse at room temperature for 45 minutes followed by addition of 5 μL of SUPERase-In RNase inhibitor (ThermoFisher Scientific) to halt the reaction. Lysates were loaded onto 13.2 mL polyallomer ultracentrifuge tubes (Beckman) containing a 10%-40% (w/v) linear sucrose gradient (10 mM Tris-HCl pH 7.5, 10mM $MgCl_2$, 10 mM NaCl).

After ultracentrifugation for 90 minutes at 40,000 rpm, 4°C in a Beckman Optima XPN-100 ultracentrifuge with an SW41 rotor, the fraction corresponding to the 80S monosomes was collected using a Teledyne ISCO model 160 fractionator equipped with a UA-6 UV/VIS detector.

The fraction was then used for RNA purification using the phenol/chloroform protocol. Ribosome-protected fragments (RPFs) measuring 28-32bp were separated and purified using a 15% Urea-TBE gel. After RNA extraction from the gel, RPFs were then dephosphorylated using 120U T4 polynucleotide kinase (New England Biolabs #M0201S) and 20U of SUPERase-InTM Rnase inhibitor in T4 PNK polynucleotide kinase buffer (New England

Biolabs) at 37 °C for 1 hour followed by 10 minutes incubation at 70 °C to inactivate the enzymes. After RPFs purification, the libraries were prepared as previously described (13). Experiments were performed in triplicate.

Ribosome profiling data analysis. Libraries from E14.5 brain and spinal cord tissues of Taiwanese mouse embryos were sequenced at the CIBIO NGS facility of the University of Trento, Italy, using an Illumina NovaSeq6000 system.

Reads clipping and trimming. Trimming of reads was performed with Cutadapt (v4.1) (14). The first three nucleotides (5' end) were trimmed and the adapter sequence of 15 As was removed (3'end). Reads shorter than 15 nucleotides were discarded. Maximum error rate and minimum overlap parameters were set at 0.15 and 10, respectively.

Reads alignment. Reads mapping on the collection of Mus musculus rRNAs (from the SILVA rRNA database, release 119) and tRNAs (from the Genomic tRNA database: gtrnadb.ucsc.edu/) were removed. The remaining reads were mapped on the mouse genome (using the Gencode M22 annotations ensembl 97) allowing a maximum of 5 multiple alignments for each read. All alignments were performed with STAR (v2.5.3a) employing default settings. Reads mapping on the same position with respect to transcript coordinates were then removed before proceeding with positional analyses. The identification of reads P-site position on the transcript was performed using riboWaltz (15). Duplicated reads mapping on the same position with respect to transcript coordinates were also removed.

Positional analysis. After having identified the P-site position of reads on the transcripts, riboWaltz was used to inspect the good quality of the ribosome profiling libraries. The read

length distribution of ribosome footprints was computed by averaging replicas of each condition (SMA and controls).

RiboWaltz was used to inspect the trinucleotide periodicity along the coding sequence and investigate potential differences in the localization of the ribosomes between control and SMA mice. Overlaid meta-profiles were computed at single nucleotide resolution, based on P-site position of ribosomes. To compare the meta-profiles, we displayed the frequency of the signal around the translation initiation and translation termination sites, so that the area under each meta-profile (composed by the portion around the start codon and the portion around the stop codon) is equal to one. Meta-profiles were computed on all protein-coding transcripts. Stars are reported on nucleotide positions with significant differences between SMA and control signal, based on pair-end T-test (* P-value < 0.05). Genes with signal lower than a specified threshold were filtered out. This specific threshold value was computed for each replicate as it follows. First, genes with zero counts for all the replicates were removed from the gene count table. We computed fragments per kilobase of transcript per million mapped reads (*fpkm)* (with the rpkm function provided within the edgeR package (16-18) values using as gene length parameter the length of each gene (i.e., the union of the isoforms exons) retrieved from the GTF file used during the alignment step previously described.

We then computed the 80th quantile of the *fpkm* distribution for each replicate and used the resulting values as cut-off in the filtering step. Genes with *fpkm* values below the threshold values for all the replicates of a condition (control or SMA) were filtered out, resulting in a total amount of 10481 genes. A multidimensional scaling plot was computed using *fpkm* values of genes (n=10481) to display the differences between the expression values of different replicates in two dimensions.

Normalization among replicates was performed with the trimmed mean of M-values normalization method (TMM) implemented in the edgeR Bioconductor package. Pairwise

differential analyses (control vs SMA) were performed independently for each tissue. Significantly differential genes were defined by the following cut-off values: *cpm_thr*=0.05, *log_2FC*_thr=0.5 and *pval_thr*=0.05.

Ingenuity Pathway Analysis (IPA)

The IPA software was used to analyse the differentially expressed genes (DEGs) dataset obtained from ribosome profiling experiments of E14.5 brain and spinal cord and to reanalyse the recently published proteomic dataset from brain and spinal cord of Taiwanese mouse embryos at E14.5 (19).

DEGs from ribosome profiling from brain and spinal cord were integrated and inputted into the IPA application (Ingenuity System, Silicon Valley, CA), applying log_2FC_thr=0.3 and pval_thr=0.05 as cut-off values. To identify affected canonical pathways, IPA assigns input gene IDs to their equivalent entries within the Ingenuity's Knowledge Database, thereby enabling accompanying mapping of each input gene ID against a curated library of accompanying molecular interactions, pathways, and targets reported in the literature. As output, IPA not only calculates the ratio of the number of molecules in the dataset per pathway to the total number of molecules assigned to that pathway, but also through a weighted calculation accounting for pathway size, molecule hierarchy and total number of interactions constituting the pathway, reports upon the likelihood of the entire pathway itself being inhibited or activated. We identified the top 10 most affected canonical pathways represented from our ribosome profiling dataset, including the cilium assembly pathway. We therefore selected the specific molecules IPA ascribed to this pathway (20 genes in total) and we performed a deeper enrichment analysis not restricted to the Ingenuity's Knowledge Database. From this analysis, IPA revealed the top two functional terms showing a strong enrichment in cilia (formation of cilia, pval=$5.02E^{-14}$ and assembly of non-motile cilium, pval=$1.60E^{-10}$). The directionality of

the enrichment was predicted by applying an overlay of the identified functional terms and the log_2FC and p-value of the genes identified in the canonical pathway.

To investigate whether signalling pathways downstream of primary cilia were affected, we performed a targeted analysis of canonical pathways using the proteomic dataset that compared the proteome of the CNS (brain and spinal cord) of control and SMA mouse embryos at E14.5 (19). Proteins were identified by containing equal or > two unique peptides. The ratios of SMA versus control abundance for each protein in both tissues were calculated and used as input for further analysis. IPA was employed to perform an enrichment analysis on our dataset, targeting specific signalling pathways known to be regulated by primary cilia (20, 21), including Sonic Hedgehog, Wnt, mTOR, TGFbeta, Notch, Hippo and NF-kB pathways. This targeted analysis allowed us to identify the dysregulated proteins present in our dataset that were ascribed to these pathways. As described above, we determined the directionality of the dysregulation, by applying an overlay of the canonical pathways with the change values of the identified proteins.

Supplemental material: figures

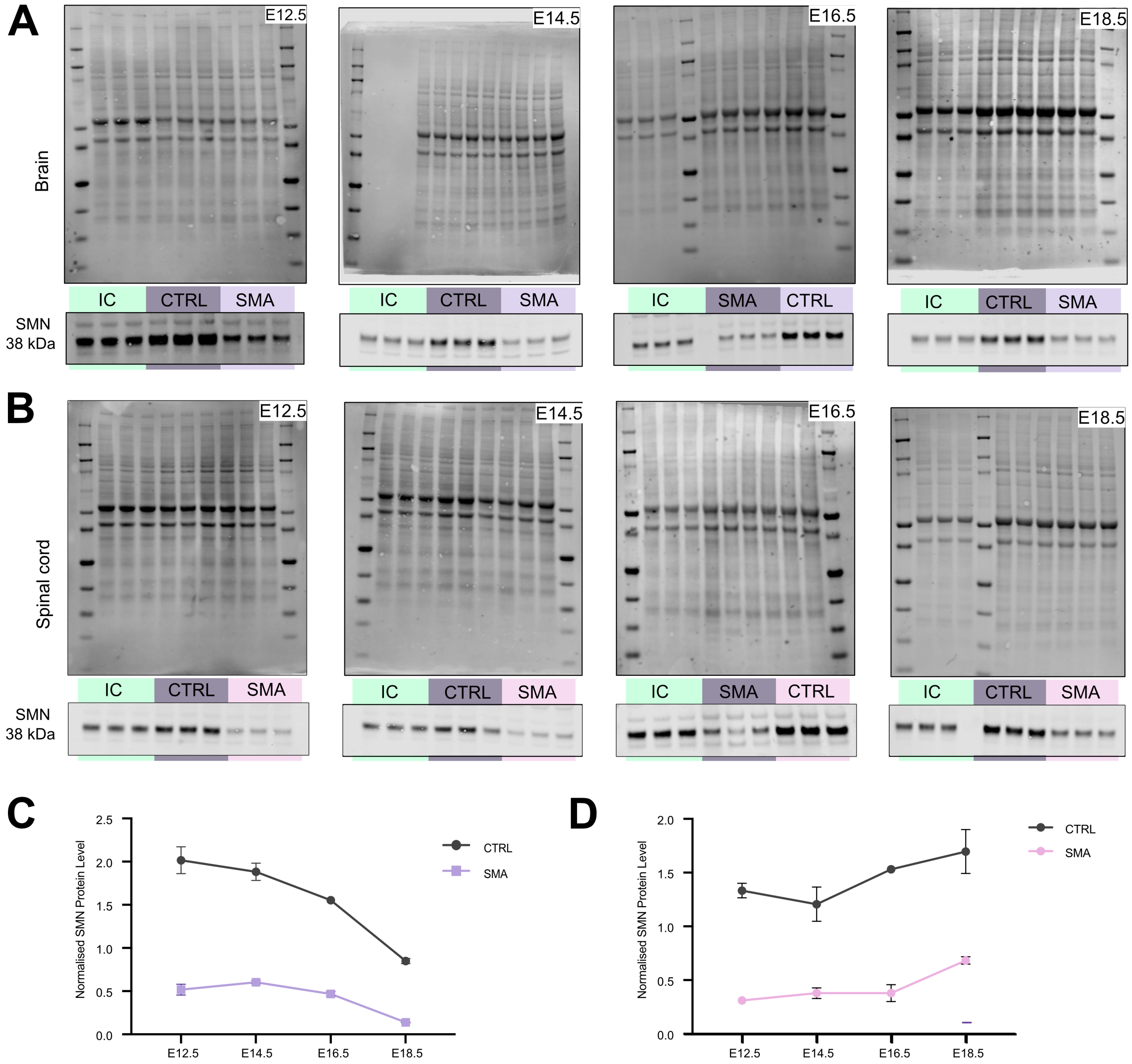

Supplementary Figure 1. Longitudinal comparison of SMN protein levels during prenatal development of brain and spinal cord. (A) Immunoblot of total protein stain and staining against SMN protein from brain and **(B)** spinal cord of controls and SMA Taiwanese mouse embryos at E12.5, E14.5, E16.5 and E18.5. Total protein stain was used to normalise total quantity of the protein for each sample. Each lane represents one embryo. N=3 for control and SMA. Internal control samples (IC) were run in triplicate to account for variability across different blots. **(C-D)** Mixed-effect linear model of SMN protein levels in the **(C)** brain and **(D)** spinal cord of controls and SMA Taiwanese mouse embryos at E12.5, E14.5, E16.5 and E18.5.

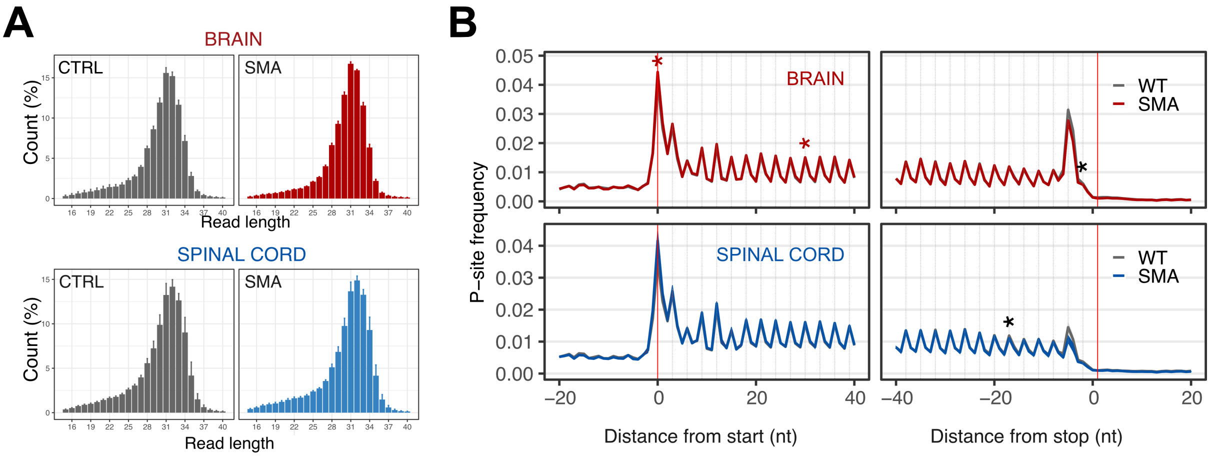

Supplementary Figure 2. Quality assessment of CNS libraries. (A) Distribution analysis of the lengths of ribosome-protected fragments in E14.5 brain and spinal cord from control and SMA samples. **(B)** Trinucleotide periodicity along the coding sequences in E14.5 brain and spinal cord from control and SMA samples. Asterisks indicate nucleotide positions with significant differences between SMA and control signal, based on pair-end T-test. *p-value < 0.05.

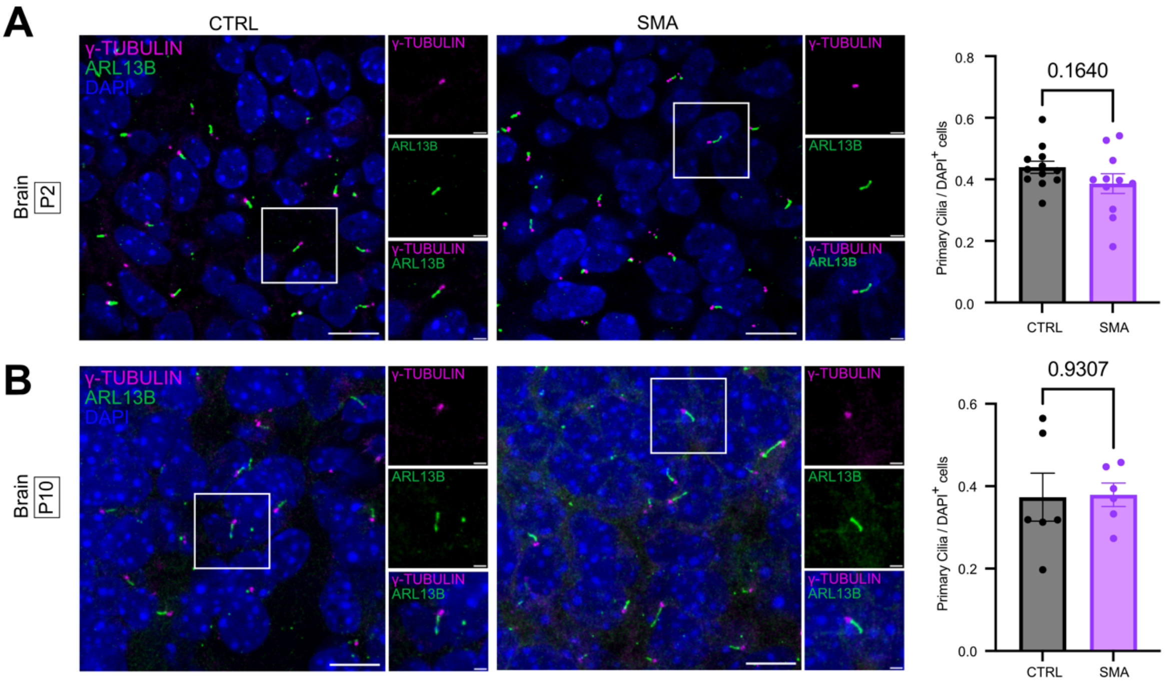

Supplementary Figure 3. No difference in primary cilia density in the hippocampus of P2 and P10 control and SMA mice. (A-B) Representative confocal images and quantification of primary cilia density in the brain of **(A)** P2 and **(B)** P10 control and SMA mice. Quantification at both timepoints did not reveal any differences between genotypes. Coronal paraffin sections, 10 µm thickness, scale bar 10 µm, zoom 2 µm. N= 12 mice for control and 11 for SMA at P2; N= 6 for control and 6 for SMA at p10. Unpaired t-test, scatter dot plot, mean with SEM. One datapoint corresponds to the average values from three sections per mouse.

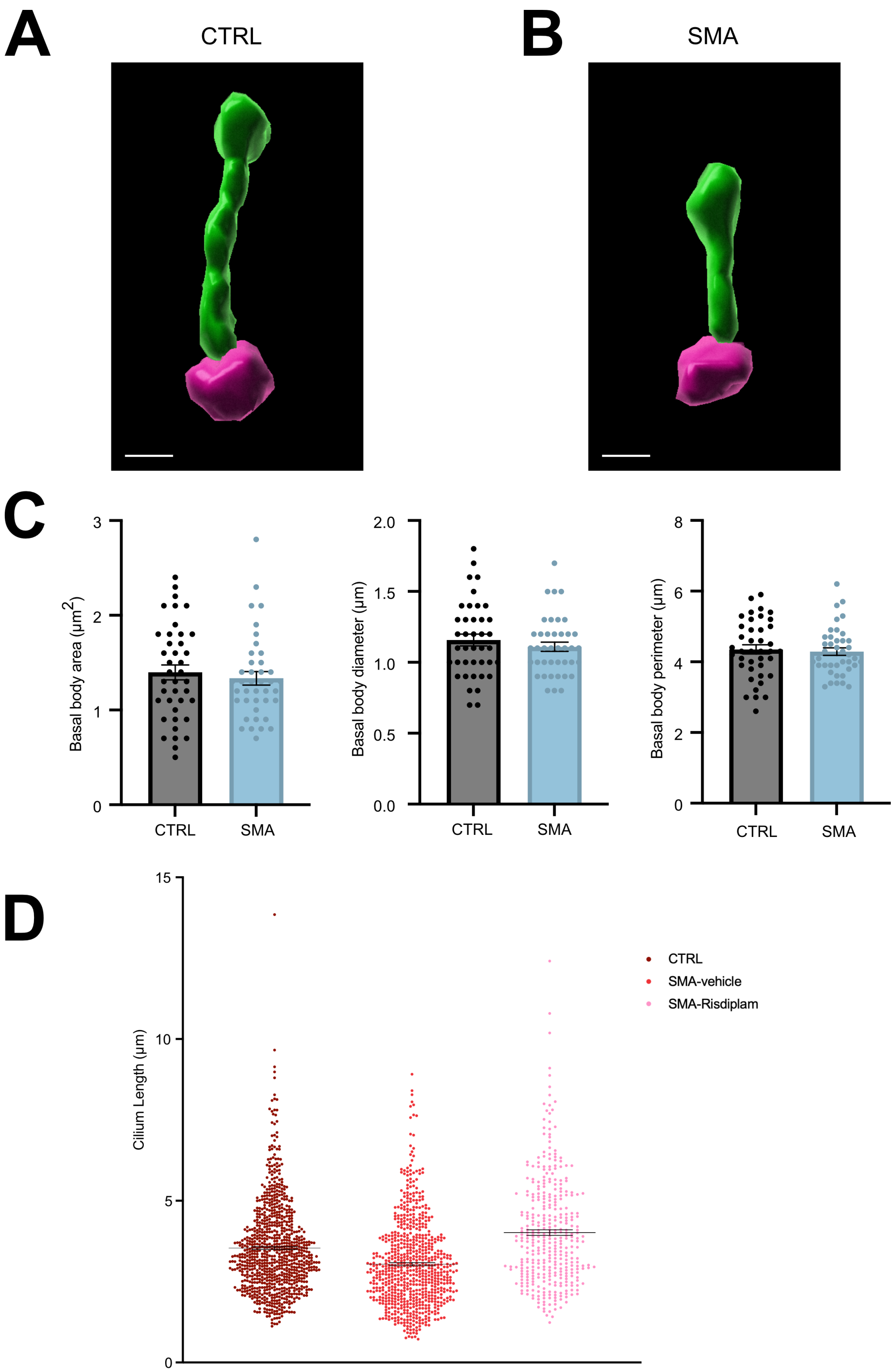
A
CTRL
B
SMA
C
Basal body area (µm2)
Basal body diameter (µm)
Basal body perimeter (µm)
CTRL
SMA
D
Cilium Length (µm)
CTRL
SMA-vehicle
SMA-Risdiplam

Supplementary Figure 4. Primary cilia morphology in vitro. (A-B) Representative 3D surface-rendered examples of primary cilia from **(A)** control and **(B)** SMA Taiwanese embryos generated from original confocal micrographs using Imaris software. Scale bar 1 μm. **(C)** Quantification of the area, diameter and perimeter of primary cilia basal bodies. The analysis was performed using Fiji/ImageJ on confocal images of hippocampal cell cultures stained for primary cilia as in Figure 4. Unpaired t-test, scatter dot plot, mean with SEM. One datapoint corresponds to one cilium. **(D)** Individual cilium length in hippocampal cell culture. Scatter plot showing the length of individual primary cilia analysed in hippocampal cell culture from controls, SMA-vehicle/untreated), and SMA-risdiplam (treated). One datapoint corresponds to one cilium. Summary of quantitative parameters is shown in Table 2. Individual values have been averaged per embryo and shown in Figure 4G.

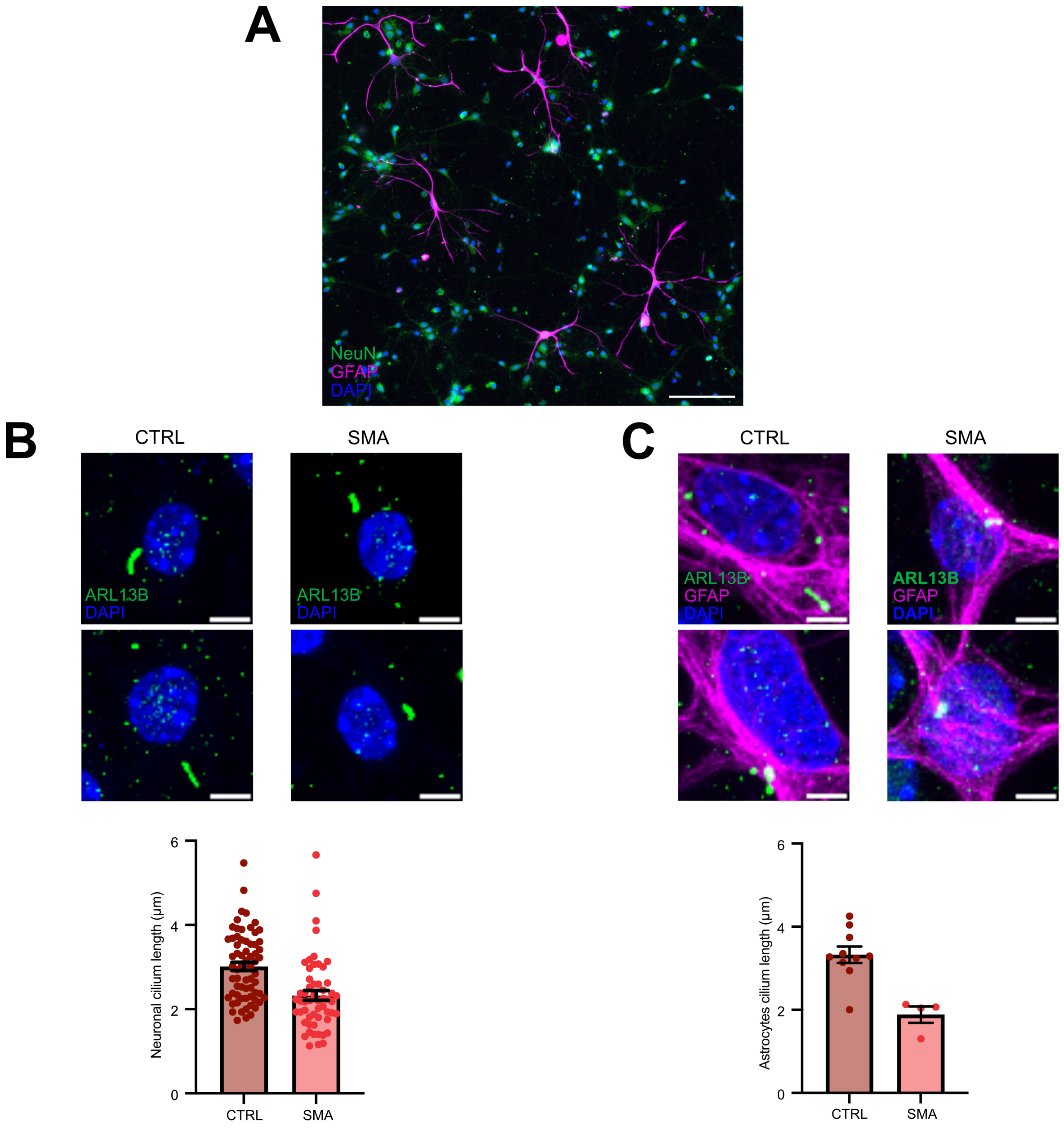

Supplementary Figure 5. Hippocampal cell culture contains neurons and astrocytes, both expressing primary cilia. (A) Immunocytochemistry on hippocampal cell culture shows the presence of neurons, labelled with NeuN (green), and astrocytes, labelled with GFAP (magenta). Representative image at 20x magnification from a control sample. Scale bar 100 µm. **(B-C)** Confocal micrographs of primary cilia, labelled with ARL13B (green) expressed in **(B)** neurons and **(C)** astrocytes, in control and SMA cell cultures. Representative length of primary cilia in neurons and astrocytes (from one coverslip per genotype) is shown in the bar charts. Scale bar = 5 µm. Each datapoint represents a single cilium

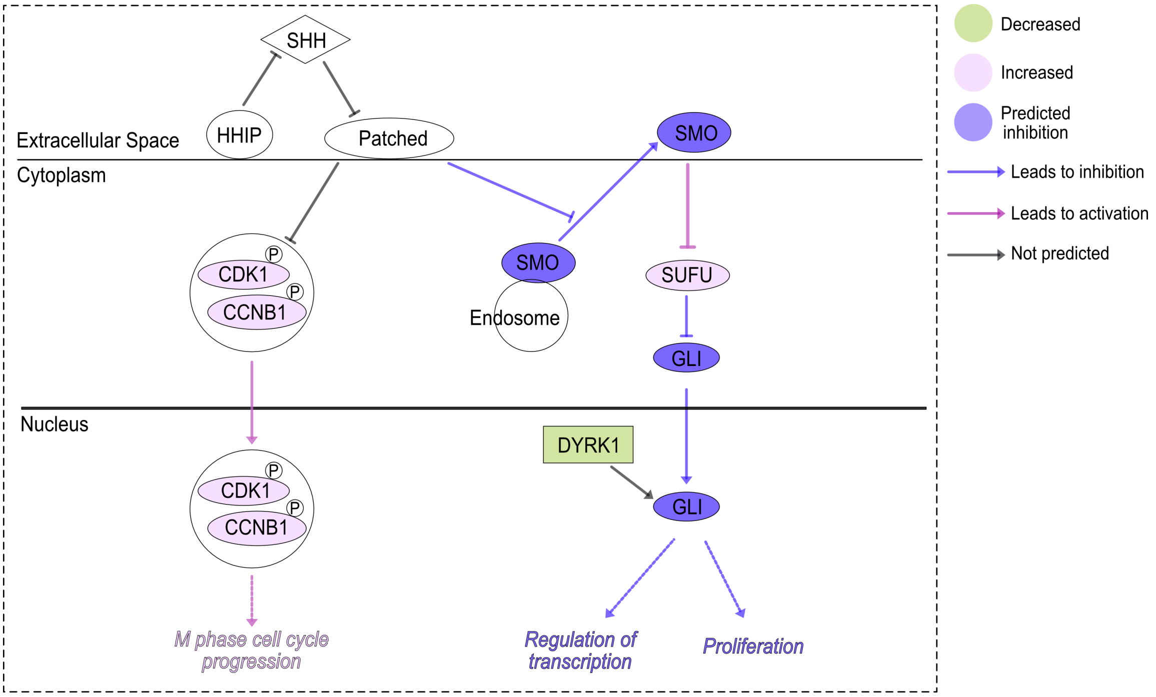

Supplementary Figure 6. Sonic Hedgehog pathway regulated by primary cilia is impaired in the CNS of E14.5 SMA mouse embryos. Schematic overview of the Sonic Hedgehog (SHH) signalling pathway. Molecules highlighted in green are downregulated, in pink are upregulated, and in purple are predicted to be inhibited in SMA mice. Purple arrows indicate inhibition; pink arrows lead to activation; grey arrows represent an effect not predicted by IPA.

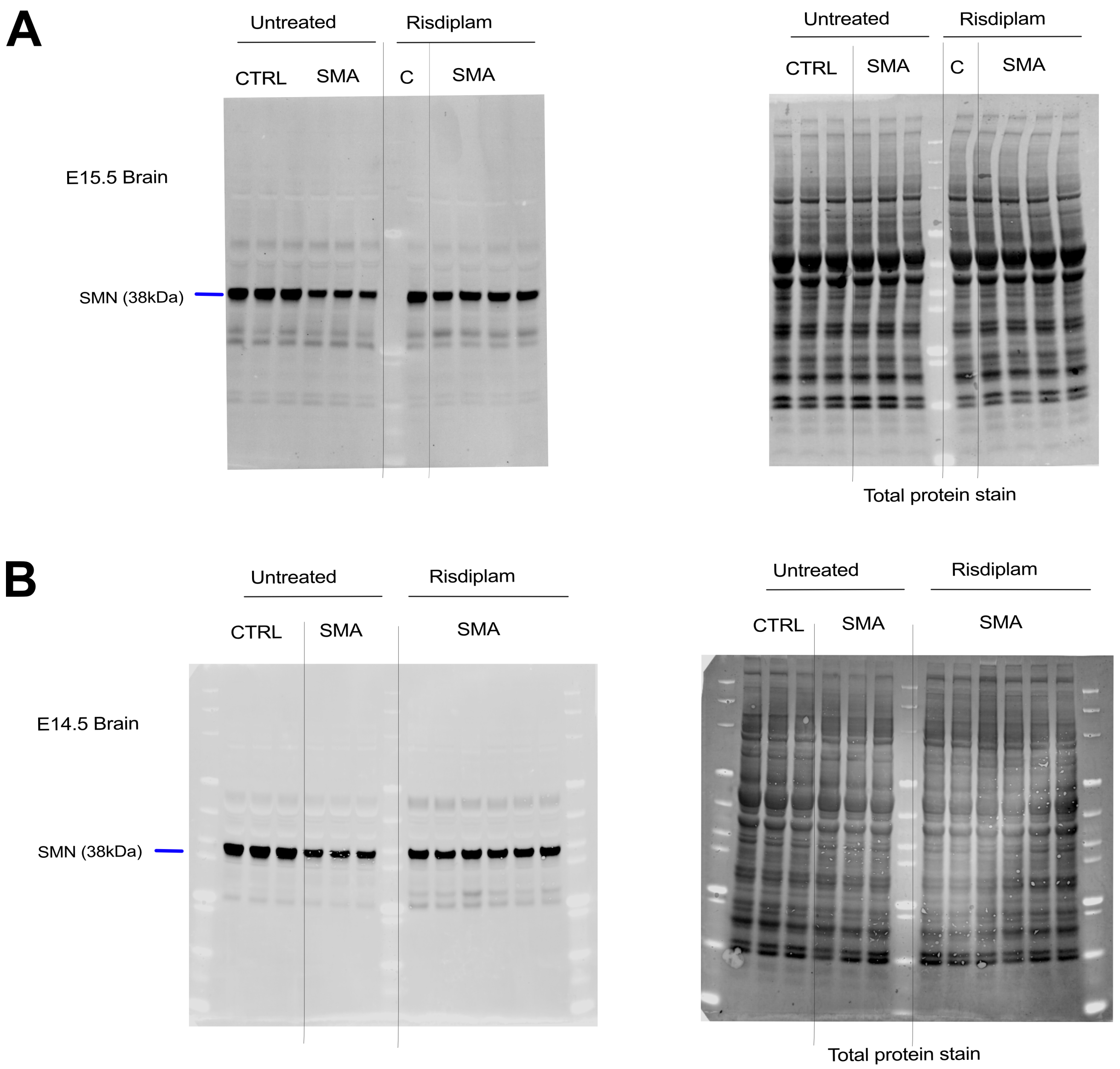

Supplementary Figure 7. SMN levels are upregulated in embryos after short duration of oral Risdiplam administration to the pregnant dam. (A) Full immunoblot from E15.5 brain tissues, corresponding Figure 6A-B. Total protein stain is used to normalise total quantity of the protein for each sample. Each lane represents one embryo. N=3 for untreated control and SMA, and N=1 and 4 for risdiplam-treated control and SMA, respectively. **(B)** Full immunoblot from E14.5 brain tissues, corresponding Figure 6D-E. Total protein stain is used to normalise protein quantify for each sample. Each lane represents one embryo. N=3 for untreated control and SMA, and N=6 for risdiplam-treated SMA. C and CTRL: healthy control.

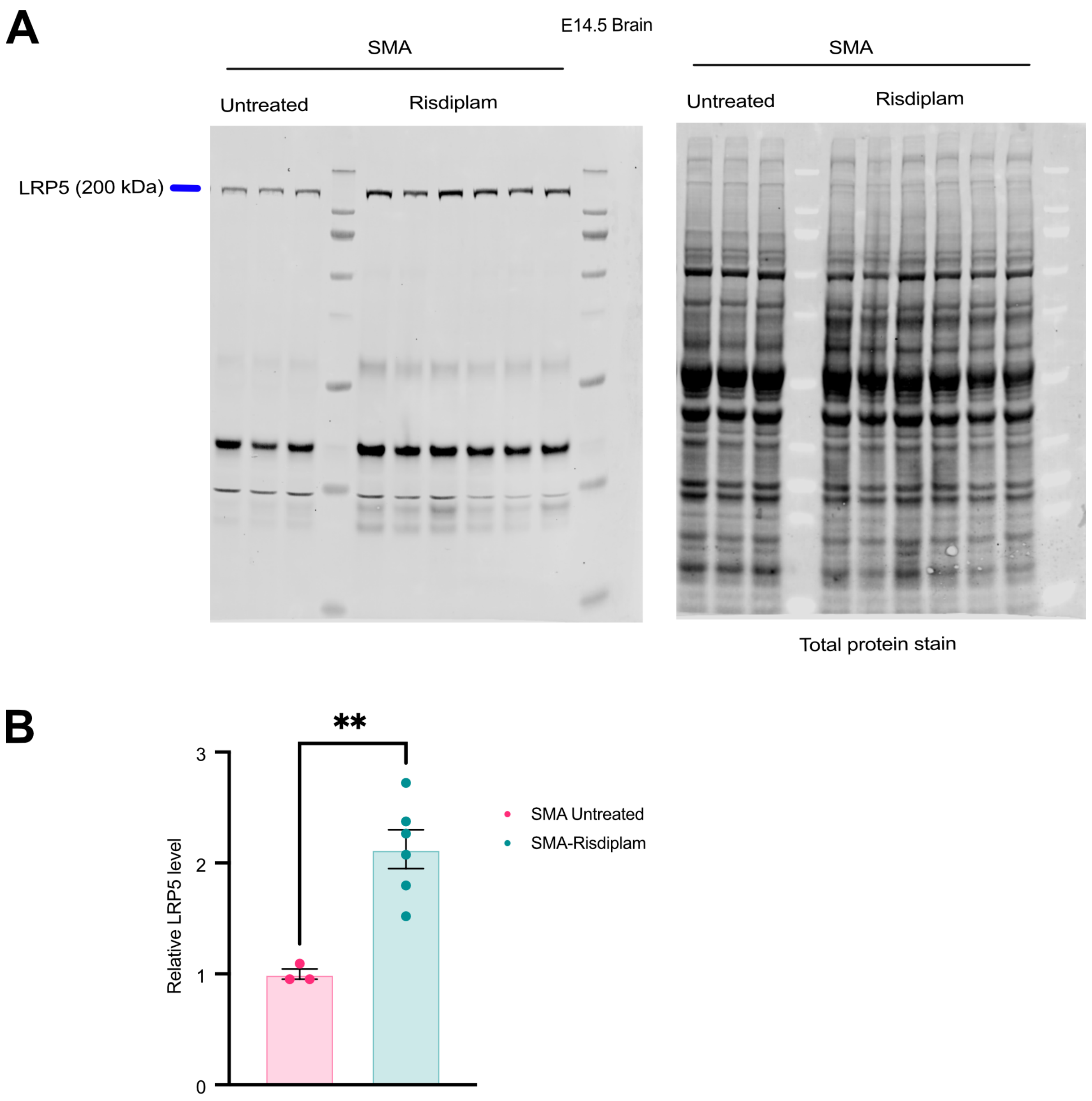

Supplementary Figure 8. Protein levels of the Wnt co-receptor, LRP5, are significantly increased in the brain of E14.5 SMA mouse embryos following in utero risdiplam treatment. (A) Immunoblot from E14.5 brain tissues. Total protein stain was used to normalise protein levels. Each lane represents one embryo. N=3 for untreated SMA, and N=6 for risdiplam-treated SMA. (B) Quantification of relative LRP5 protein levels from (A). One datapoint corresponds to one embryo. Unpaired t-test, scatter dot plot, mean with SEM. **p-value $\leq$ 0.01.

Supplemental material: table

Supplementary Table 1. Top ten dysregulated proteins in the CNS of E14.5 Taiwanese mouse embryos associated with additional signalling pathways known to be regulated by primary cilia as identified by IPA

Notch		Hippo		NF-kB	
**Protein**	**FC**	**Protein**	**FC**	**Protein**	**FC**
NUMBL	1.201	CD44	-1.305	AKT2	-2.82
RBPJ	1.122	PPP2R5B	-1.194	PLCG2	-1.348
DTX3	1.12	DLG2	-1.139	TGFBR3	-1.315
CNTN1	-1.08	TEAD2	1.111	TRAF6	-1.308
DTX2	1.068	PARD3	-1.102	RRAS	-1.207
NCSTN	1.047	TEAD1	1.097	TDP2	1.149
JAG1	1.045	FAT4	-1.097	EGF	-1.114
ADAM17	-1.03	FRMD6	-1.097	RAP2B	-1.114
NOTCH3	1.022	SKP2	-1.095	HDAC2	1.105
NOTCH2	1.012	PPP2R3A	-1.084	PIK3C2A	1.105

In continuation of Table 3, this table details protein symbols and their respective fold change for individual proteins associated with Notch, Hippo or NF-kB signaling pathways.

References:

1. Tunster SJ. Genetic sex determination of mice by simplex PCR. *Biology of Sex Differences.* 2017;8(1).
2. Hsieh-Li HM, Chang J-G, Jong Y-J, Wu M-H, Wang NM, Tsai CH, et al. A mouse model for spinal muscular atrophy. *Nature Genetics.* 2000;24(1):66-70.
3. Riessland M, Ackermann B, Förster A, Jakubik M, Hauke J, Garbes L, et al. SAHA ameliorates the SMA phenotype in two mouse models for spinal muscular atrophy. *Human Molecular Genetics.* 2010;19(8):1492-506.
4. Signoria I, van der Pol WL, and Groen EJN. Innovating spinal muscular atrophy models in the therapeutic era. *Dis Model Mech.* 2023;16(9).
5. Richardson L, Venkataraman S, Stevenson P, Yang Y, Moss J, Graham L, et al. EMAGE mouse embryo spatial gene expression database: 2014 update. *Nucleic Acids Res.* 2014;42(Database issue):D835-44.
6. Poirier A, Weetall M, Heinig K, Bucheli F, Schoenlein K, Alsenz J, et al. Risdiplam distributes and increases <scp>SMN</scp> protein in both the central nervous system and peripheral organs. *Pharmacology Research & Perspectives.* 2018;6(6):e00447.
7. Groen EJN, Perenthaler E, Courtney NL, Jordan CY, Shorrock HK, van der Hoorn D, et al. Temporal and tissue-specific variability of SMN protein levels in mouse models of spinal muscular atrophy. *Hum Mol Genet.* 2018;27(16):2851-62.
8. Bates D, Mächler M, Bolker BM, and Walker SC. Fitting Linear Mixed-Effects Models Using lme4. *J Stat Softw.* 2015;67(1):1-48.
9. Brewer GJ, Torricelli JR, Evege EK, and Price PJ. Optimized survival of hippocampal neurons in B27-supplemented Neurobasal, a new serum-free medium combination. *J Neurosci Res.* 1993;35(5):567-76.

10. Zhou H, Janghra N, Mitrpant C, Dickinson RL, Anthony K, Price L, et al. A Novel Morpholino Oligomer Targeting ISS-N1 Improves Rescue of Severe Spinal Muscular Atrophy Transgenic Mice. *Human Gene Therapy.* 2013;24(3):331-42.

11. Ruggiu M, McGovern VL, Lotti F, Saieva L, Li DK, Kariya S, et al. A role for SMN exon 7 splicing in the selective vulnerability of motor neurons in spinal muscular atrophy. *Mol Cell Biol.* 2012;32(1):126-38.

12. Bernabò P, Tebaldi T, Groen EJN, Lane FM, Perenthaler E, Mattedi F, et al. In Vivo Translatome Profiling in Spinal Muscular Atrophy Reveals a Role for SMN Protein in Ribosome Biology. *Cell Reports.* 2017;21(4):953-65.

13. Lauria F, Bernabò P, Tebaldi T, Groen EJN, Perenthaler E, Maniscalco F, et al. SMN-primed ribosomes modulate the translation of transcripts related to spinal muscular atrophy. *Nature Cell Biology.* 2020;22(10):1239-51.

14. Martin M. Cutadapt removes adapter sequences from high-throughput sequencing reads. *2011.* 2011;17(1):3.

15. Lauria F, Tebaldi T, Bernabò P, Groen EJN, Gillingwater TH, and Viero G. riboWaltz: Optimization of ribosome P-site positioning in ribosome profiling data. *PLOS Computational Biology.* 2018;14(8):e1006169.

16. Chen Y, Lun ATL, and Smyth GK. From reads to genes to pathways: differential expression analysis of RNA-Seq experiments using Rsubread and the edgeR quasi-likelihood pipeline. *F1000Research.* 2016;5:1438.

17. McCarthy DJ, Chen Y, and Smyth GK. Differential expression analysis of multifactor RNA-Seq experiments with respect to biological variation. *Nucleic Acids Research.* 2012;40(10):4288-97.

18. Robinson MD, McCarthy DJ, and Smyth GK. <tt>edgeR</tt>: a Bioconductor package for differential expression analysis of digital gene expression data. *Bioinformatics.* 2010;26(1):139-40.

19. Motyl AAL, Faller KME, Groen EJN, Kline RA, Eaton SL, Ledahawsky LM, et al. Pre-natal manifestation of systemic developmental abnormalities in spinal muscular atrophy. *Hum Mol Genet.* 2020;29(16):2674-83.

20. Anvarian Z, Mykytyn K, Mukhopadhyay S, Pedersen LB, and Christensen ST. Cellular signalling by primary cilia in development, organ function and disease. *Nature Reviews Nephrology.* 2019;15(4):199-219.

21. Mill P, Christensen ST, and Pedersen LB. Primary cilia as dynamic and diverse signalling hubs in development and disease. *Nature Reviews Genetics.* 2023;24(7):421-41.
