# Supplementary figures and images for: Prenatal SMN-dependent defects in translation uncover reversible primary cilia phenotypes in spinal muscular atrophy
Source: JCI Insight. 2025 Sep 9;10(20):e192835. doi: 10.1172/jci.insight.192835 (PMC12581668; doi:10.1172/jci.insight.192835)

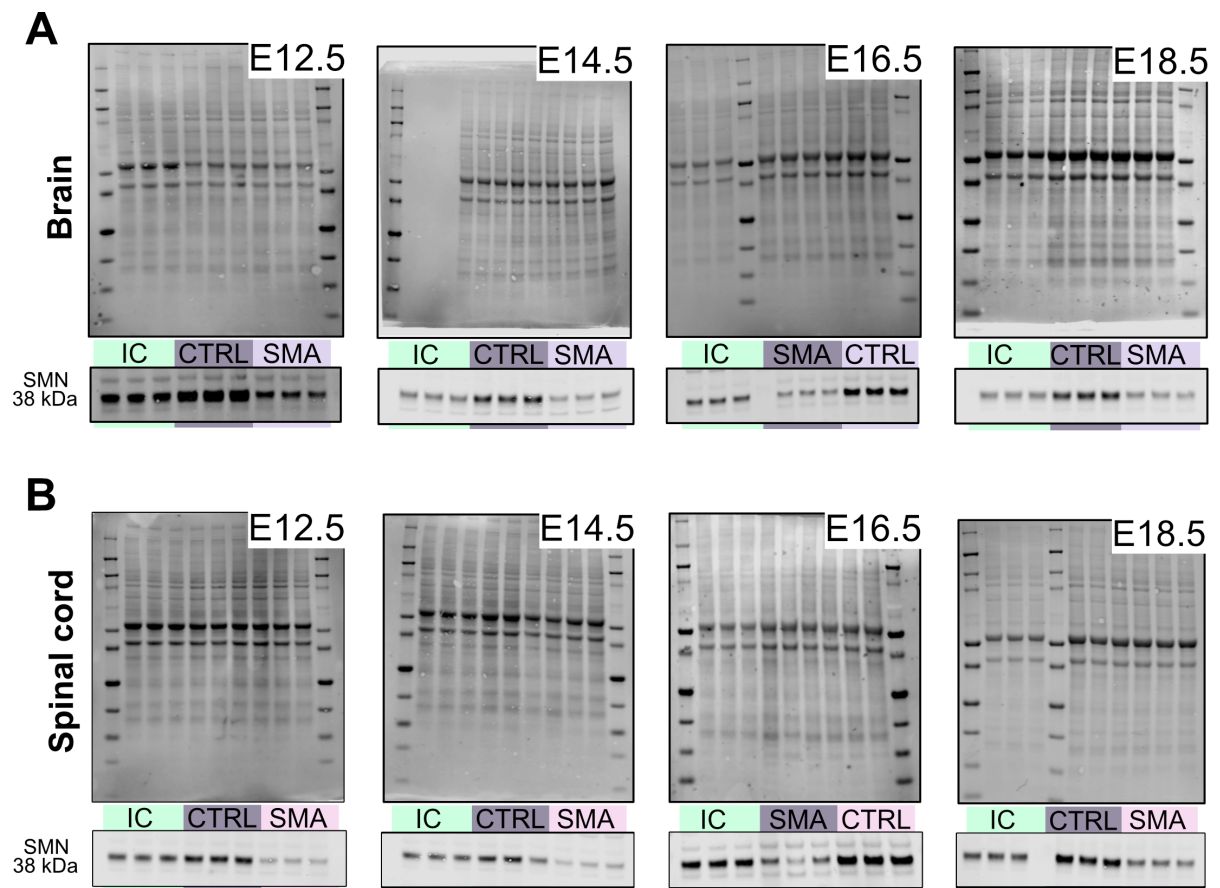

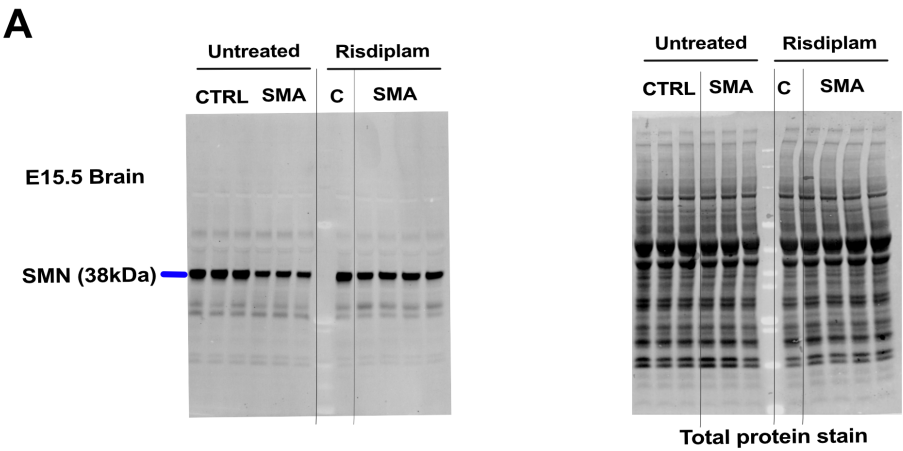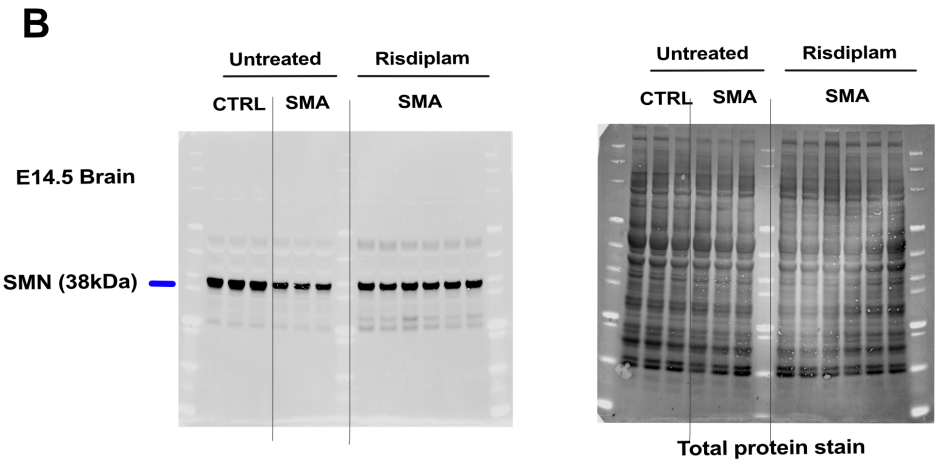

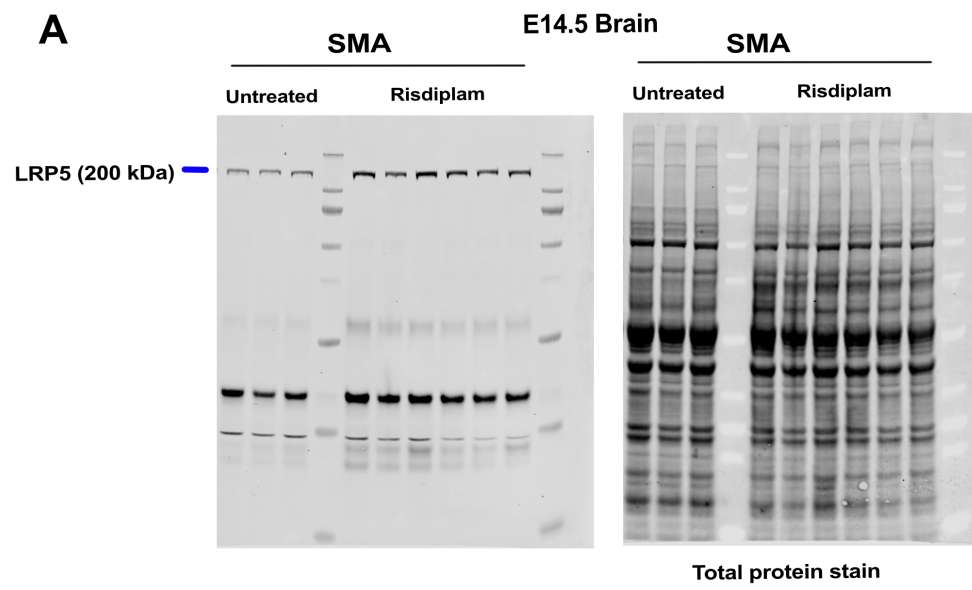

Supplement: Unedited blot and gel images [file jciinsight-10-192835-s143.pdf]
